# De-identified Bayesian personal identity matching for privacy-preserving record linkage despite errors: development and validation

**DOI:** 10.1186/s12911-023-02176-6

**Published:** 2023-05-05

**Authors:** Rudolf N. Cardinal, Anna Moore, Martin Burchell, Jonathan R. Lewis

**Affiliations:** 1 Department of Psychiatry, University of Cambridge, Cambridge Biomedical Campus, Clifford Allbutt Building, Bay 13, Cambridge, CB2 0AH UK; 2 Cambridgeshire & Peterborough NHS Foundation Trust, Fulbourn Hospital, Cambridge, CB21 5EF UK

**Keywords:** Bayesian probabilistic linkage, De-identification, Pseudonymisation, Electronic health records, Electronic medical records, Electronic patient records, Identity matching, Open-source software, Privacy-preserving record linkage, Psychiatry, Mental health

## Abstract

**Background:**

Epidemiological research may require linkage of information from multiple organizations. This can bring two problems: (1) the information governance desirability of linkage without sharing direct identifiers, and (2) a requirement to link databases without a common person-unique identifier.

**Methods:**

We develop a Bayesian matching technique to solve both. We provide an open-source software implementation capable of de-identified probabilistic matching despite discrepancies, via fuzzy representations and complete mismatches, plus de-identified deterministic matching if required. We validate the technique by testing linkage between multiple medical records systems in a UK National Health Service Trust, examining the effects of decision thresholds on linkage accuracy. We report demographic factors associated with correct linkage.

**Results:**

The system supports dates of birth (DOBs), forenames, surnames, three-state gender, and UK postcodes. Fuzzy representations are supported for all except gender, and there is support for additional transformations, such as accent misrepresentation, variation for multi-part surnames, and name re-ordering. Calculated log odds predicted a proband’s presence in the sample database with an area under the receiver operating curve of 0.997–0.999 for non-self database comparisons. Log odds were converted to a decision via a consideration threshold *θ* and a leader advantage threshold *δ*. Defaults were chosen to penalize misidentification 20-fold versus linkage failure. By default, complete DOB mismatches were disallowed for computational efficiency. At these settings, for non-self database comparisons, the mean probability of a proband being correctly declared to be in the sample was 0.965 (range 0.931–0.994), and the misidentification rate was 0.00249 (range 0.00123–0.00429). Correct linkage was positively associated with male gender, Black or mixed ethnicity, and the presence of diagnostic codes for severe mental illnesses or other mental disorders, and negatively associated with birth year, unknown ethnicity, residential area deprivation, and presence of a pseudopostcode (e.g. indicating homelessness). Accuracy rates would be improved further if person-unique identifiers were also used, as supported by the software. Our two largest databases were linked in 44 min via an interpreted programming language.

**Conclusions:**

Fully de-identified matching with high accuracy is feasible without a person-unique identifier and appropriate software is freely available.

**Supplementary Information:**

The online version contains supplementary material available at 10.1186/s12911-023-02176-6.

## Background

### Motivation

**Fig. 1:**
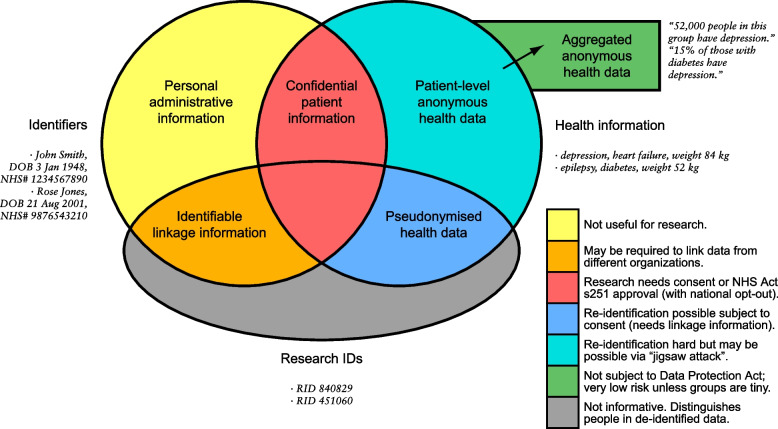
Types of information found in medical records and corresponding research databases, with UK legal status. Typically, researchers seek to operate with pseudonymised or anonymised health data, prior to publishing aggregated anonymous data. A challenge is to produce linked pseudonymised/anonymous health data from multiple organizations without exchanging identifying information. (All examples are fictional.) From [3]

A common problem in epidemiological research involving health data is the linkage of information about the same person from multiple sources. This brings two significant problems: information governance (IG) constraints and the potential lack of a common unique identifier. Medical records contain health information (in United Kingdom [UK] terminology, “patient information”) [1], plus identifiers (IDs) such as names and dates of birth (DOB). Under UK law, the combination of these two represents confidential patient information [1]. Identifiers alone also represent personal data [2], but of a more limited kind; we term this personal administrative information. The goal for research is often to de-identify health data, removing direct identifiers and tagging each person’s record with a research ID to create pseudonymised health data (in principle re-identifiable given the mapping between research ID and direct identifiers), or removing all identifiers to create anonymous health data (Fig. 1).

**Fig. 2:**
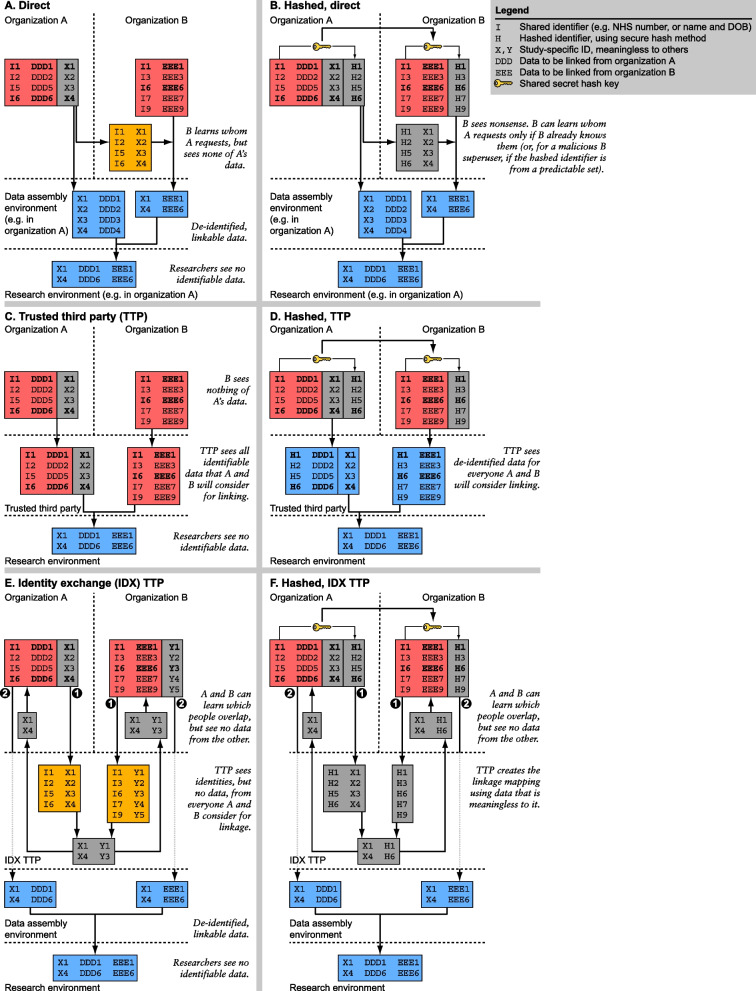
Linkage techniques between two hypothetical organizations, with and without the use of direct identifiers. The primary objective is to link data from two (e.g. healthcare) organizations A and B, so that information about a given person can be related. The secondary objective is to do so for research in a way that researchers cannot see identifiable data. All methods shown achieve these objectives. The tertiary objective is to minimize or eliminate handling of direct identifiers during the process of linkage, and more generally to minimize the ability of any participating organization to learn facts about any person that they did not already know. Information is colour-coded according to Fig. 1. All methods using de-identified linkage (right-hand column) require the ability to match people without the use of direct (plaintext) identifiers. This is simple with a shared unique identifier (e.g. NHS number) but more difficult without one, a technique developed in the present study. **A** *Direct linkage.* Organization A sends identity information (I) but not health information to B, tagging every person with a research ID (X) of no meaning to anyone else. A’s data, and the subset of B’s for people who match, are de-identified and linked for research. **B** *Hashed direct linkage.* If A and B share a secret hash key, they can reproduce this process but instead of using direct identifiers (I), they can use an irreversibly hashed version (H). **C** *Trusted third-party (TTP) linkage.* If A and B share a TTP, that TTP can perform linkage using identifiable data without B learning whom A requests, before de-identifying the linked data for research. **D** *Hashed TTP linkage.* As before, sharing a hash key enables the TTP to operate without direct identifiers. **E** *Identity exchange (IDX) TTP linkage.* In this more complex scheme, the TTP is used only to exchange identity information, without having to hold health information. Identity linkage (➊) occurs before de-identified health data linkage (➋). **F** *Hashed IDX TTP linkage.* The process can be improved further by hashing

When two organizations A and B communicate to link health data, with the ultimate purpose of creating de-identified data for research, further IG challenges arise. The simplest technique would be for A to send its identifiable data wholesale to B, so that B can identify patients common to A and B, and prepare a de-identified data set. However, this provides B with a great deal of information about A’s patients, which is undesirable. Such work is likely to require special approvals, e.g. in the UK under Section 251 of the National Health Service (NHS) Act [1], and typically involves unnecessary exchange of information. Instead, a variety of linkage strategies are possible, such as linkage using identifiers only, the use of a trusted third party, a combination of these strategies (Fig. 2A,C,E), or closely related methods [4]. Nevertheless, these still involve the exchange of identifiers, which may require special regulatory approval.

A more desirable process would be to exchange irreversibly encrypted or hashed identity information for linkage, removing the need for identifiable data to leave any organization (Fig. 2B,D,F). Such a process is technically trivial if the two organizations share a common unique patient identifier (such as the NHS number), requiring only the exchange of a shared cryptographic key (Fig. 2).

Linkage is substantially harder between organizations that do not share a common identifier. If the only shared identifiers are non-unique ones such as names, DOBs, or addresses, then linkage must deal with the possibility of non-unique matches. If errors are present, the process becomes harder.

### Existing linkage techniques

Conceptually, probabilistic linkage systems [5] address the problem of matching records based on non-unique and potentially missing identifiers, and many also address the problem of partially inaccurate or corrupted identifiers (e.g. names with typographical errors) via some form of inexact or “fuzzy” comparison. Some other linkage systems use simple deterministic linkage [6]. Rule-based fuzzy matching, conceptually close to fully deterministic linkage, has been used to link health and education data [4]. Linkage techniques using machine learning also exist [5].

Probability-based techniques centre around Bayes’ theorem [7]. For a given pair of records to be compared, Bayesian linkage techniques typically begin with a prior probability of a person match and modify this according to identifier agreement (equality) or disagreement (inequality). However, the detail or granularity of this approach varies. A basic Bayesian approach uses probabilities relating to identifier types, also called fields, components, characteristics, or items (e.g. “the probability of the other record having the same forename if the two records are/are not the same person” for the item “forename”), recognizing that the information conveyed by different items differs (e.g. DOB carries more information than sex/gender). A more accurate method is to use probabilities relating to specific identifiers (e.g. “the probability of the other record also having the forename John if the two records are/are not the same person”), recognizing, for example, that rare forenames carry more information than common forenames [8–10]. This approach was developed by Newcombe et al. [8] though was not described explicitly as Bayesian at the time [8, 11, 12], and was formalized by Fellegi and Sunter [9]. Even within such a system, the level of detail in the implementation varies, such as whether probabilities are estimated for individual surnames or for groups of surnames [13]; some systems use a combination of item-level and identifier-specific weights [13]; and some have mischaracterized the Fellegi–Sunter approach as being item-level only [14, 15]. Explicit prior probabilities are not used in Fellegi–Sunter, but that is not relevant within its threshold-based decision system (described below). The concrete simplified Fellegi–Sunter method assumes conditional independence of items [5, 9, 16], but additional sophistication is added by recognizing that items may be conditionally dependent, e.g. that sex/gender may affect forename frequencies or the chance of a surname mismatch [5]. Bayesian methods may also be used to estimate the probabilities of identifier agreement/disagreement themselves [5, 17–20].

For practical use, probability or a related quantity must be converted to a categorical decision about linkage. In the Fellegi–Sunter method [9], pairs of records are ordered by the likelihood ratio for being a match. Two cutoffs are then set: one threshold above which a match is declared, and another threshold below which a non-match is declared. The intermediate zone of “possible links” is uncertain and might be sent for human review [5, 9, 16]. The procedure is optimal at minimizing the size of the uncertain set, given prespecified rates of type I error (false linkage) and type II error (incorrectly unlinked records) [9]. It is compatible with a two-state choice (link or not-link), simply by setting the size of the uncertain zone to zero [9]. It has been asserted that it depends on an assumption of no duplicate records [15], but the original work has no such restriction [9]. Many systems use the Fellegi–Sunter method [5], and software implementations are readily available, including a Python package based on an adapted Fellegi–Sunter model [21, 22]. Bayesian records linkage has been applied before to good effect in many contexts [23], such as for the SAIL (Secure Anonymised Information Linkage) Databank in Wales [24].

For identifiable linkage, exact identifier comparison is trivial, and a range of approaches have been used for fuzzy comparison. These include fuzzy string comparison, such as via the Levenshtein edit distance [25, 26] or via phonetic (“sounds-like”) algorithms [27, 28]. Fuzzy representations such as phonetic distillations may be compared exactly, yielding a yes/no decision about the approximate equality of the original identifier. With distance metrics, which provide a continuous measure of similarity, approximate matching in a Fellegi–Sunter system can use the metric to determine agreement versus disagreement via a cutoff [29], or as a weight to calculate evidence in between that of full agreement and disagreement [30]; more sophisticated systems examine the probability distribution by different levels of disparity [14, 29].

De-identification adds a further challenge. Existing de-identified (privacy-preserving) linkage systems typically involve comparison of hashed identifiers. The hashed representation may have relatively high information content and a low hash “collision” rate (meaning that it is very unlikely that two different identifiers hash to the same result), thus permitting only exact comparison of hashed identifiers [31, 32]. To account for source errors, “inexact” versions of the identifiers (such as phonetic representations) may therefore be incorporated as well [31, 32]. In alternative schemes, such as via Bloom filters [33], hashed identifiers may have high collision rates but represent multiple aspects (e.g. encoding all digrams, trigrams, or other *q*-grams of a string), permitting string similarity measures to be obtained from pairs of hashed values themselves, and thus inexact comparison in the de-identified domain [34–36]. Related techniques incorporate security improvements to improve on the Bloom filter method by preventing frequency attacks [36, 37]. Linkage is also possible via domain-specific data that are not intrinsically directly identifying, such as surgical procedures or operation dates [38]; we do not consider such systems here.

However, we are unaware of a previously published technique that combines identity hiding with identifier-level probability representations for probabilistic linkage of de-identified data. Phonetic techniques using algorithms such as SoundEx [39] and MetaSoundex [40] implement privacy-preserving linkage without explicit probability calculations. Some existing Bloom filter techniques [35] and related fuzzy string comparison systems [36] have used a similarity threshold to make a match decision, but again did not use explicit probability comparisons. Some Bloom filter implementations have used item-level weights via a Fellegi–Sunter approach [34], with the dice coefficient between pairs of Bloom filters (range 0–1) as the similarity metric. Other string similarity systems also use the concept of item weight [41]. However, these systems do not explicitly represent the probabilities associated with specific identifiers, or address lack of conditional independence. An R package exists for privacy-preserving record linkage [30], but its probabilistic linkage methods combine a similarity function with (empirically) conditionally independent item-level probability weights, estimated via expectation maximization [10, 42, 43] based on user-supplied starting values and the data being linked, and using the Fellegi–Sunter method with weighting for approximate matching [30]; it has been described as requiring “a significant understanding of statistical programming… for usage” [5].

### Goals

Here, we develop a system combining identifier-level probability representations with de-identified linkage. Our goals were for an easy-to-use, practical, and extensible open-source system for privacy-preserving record linkage; to support aspects of fuzzy comparison; to apply domain-specific knowledge about identifier types in common use for linkage in our context (UK healthcare), such as about name frequency and conditional dependence of some identifier types; to incorporate user-customizable frequency/error data, with sensible defaults, to make it generalizable to databases beyond those from which defaults were derived (and to validate such generalization); to incorporate user-specified priors and thus produce an absolute probability estimate of linkage; and to permit easy end-user validation against reference data. We include the ability to control frequency precision, and other techniques to limit trivial frequency attack; however, any frequency representation related to identifiers increases the possibility of malicious re-identification [37, 44], and our objective was to remove the use of direct identifiers rather than to guarantee against attack. That is, the representations used by our system are intended to pass the “reasonably likely” test [45] rather than the “motivated intruder” test [46]. This is appropriate to support de-identified linkage between organizations to create person-level de-identified data [47] for use in a trusted research environment, because such person-level data must often be assumed itself intrinsically unsafe against motivated attack [48, 49] and require statistical disclosure control [50, 51] prior to broader publication. We assume that at least one side of the linkage contains candidate records intended to represent distinct non-duplicated people, and thus that the goal is to find zero or one link for every proband from the other side (though the system could also be used to find potential duplicates via a self-linkage, discussed later). We derive a posterior probability of linkage that is then subjected to a binary decision [9], but consider also a second threshold to prevent linkage when multiple candidate records are very similar (e.g. inadvertent duplicates or genuinely similar different individuals), for situations where failure to link is preferred strongly to spurious linkage.

## Design

**Fig. 3:**
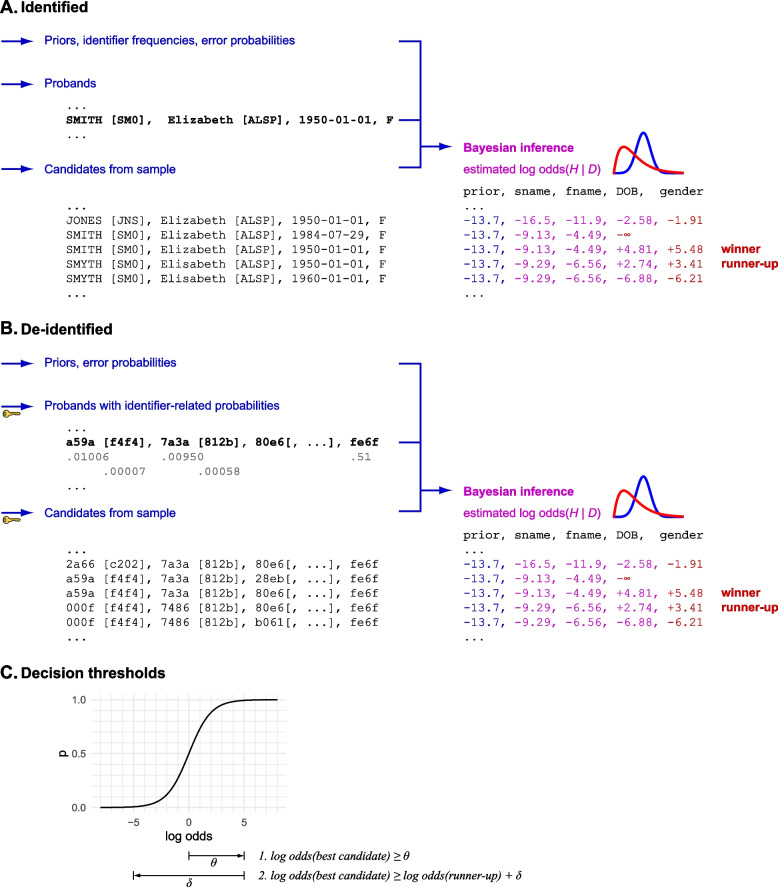
Simplified illustration of Bayesian matching. This process omits some of the methods of the full algorithm, and shows only a three-state comparison for names (name match, metaphone match, or no match). Comparison settings are as in Table 2. **A** Identified version. Calculations are shown left to right across identifiers: the log odds are updated with every successive identifier comparison. **B** De-identified version, showing identifier probabilities explicitly (e.g. the population probability of SMITH as a surname and of the corresponding metaphone SM0 arising from other surnames). Hashed identifiers are shown as four hexadecimal characters but in practice would be much longer. Some hashed representations are more complex than shown (e.g. “fuzzy” DOB representations; see text). **C** Winners are declared or not based on two decision thresholds, *θ* and *δ* (see text)

We begin by describing a specific Bayesian identity matching scheme using direct identifiers, acknowledging the potential for errors in the data (Fig. 3A). We assume that people from organization A must be matched to people in organization B. If A and B both use a common unique identifier, this problem would be trivial.

### Terminology

We define the *population* (of size *n*_*p*_) as all people of interest who could possibly be considered (e.g. the population of the UK, or of Cambridgeshire). We define the *sample* (of size *n*_*s*_ ≤ *n*_*p*_) as the group of people against whom we are matching—in our example, all those from organization B. We define the *proband* as the person of interest, from organization A, for whom a match is sought in the sample. When a specific member of the sample is under consideration and being compared to the proband, we refer to them as the *candidate*. By definition, all probands and all candidates are members of the population.

### Population and sample

Some central government databases might in theory cover their whole population, such that *n*_*s*_ = *n*_*p*_. More commonly, samples will be subsets of the population. For example, Cambridgeshire & Peterborough NHS Foundation Trust (CPFT), our test site for this work, provides health services primarily to the population of Cambridgeshire and Peterborough; its population is therefore of the order of *n*_*p*_ ≈ 1 M people. For simplicity, let us assume that this is its exclusive population (ignoring patients from farther afield). Only a proportion of that population actually have records with CPFT; let us suppose a sample of *n*_*s*_ ≈ 100,000 (in practice a large underestimate). A proband randomly drawn from Cambridgeshire would therefore have a *n*_*s*_/*n*_*p*_ = 10% chance of being in the CPFT database, and the prior chance of the proband being *any individual member* of the sample is 1/*n*_*p*_ = 10^−6^, irrespective of the sample size.

Under the assumption that the proband is randomly drawn from the population, it is easy to illustrate the irrelevance of *n*_*s*_ for the prior probability that a proband matches an individual candidate: there is a probability *n*_*s*_/*n*_*p*_ that the proband is in the sample and a probability 1 − *n*_*s*_/*n*_*p*_ that they are not, and thus a probability (1/*n*_*s*_)⋅(*n*_*s*_/*n*_*p*_) + 0⋅(1 − *n*_*s*_/*n*_*p*_) = 1/*n*_*p*_ that a given candidate from the sample is the proband. The situation would be different, of course, if the proband was more likely to come from the sample than from the population as a whole.

### Starting prior and Bayesian update

We define *H* as the hypothesis that a specific candidate is the same person as the proband. We start with the prior probability for the candidate, *P*(*H*) = 1/*n*_*p*_. We update this probability sequentially with incoming data *D* from the proband and candidate to give *P*(*H* | *D*) according to Bayes’ theorem [7], *P*(*H* | *D*) = *P*(*D* | *H*)⋅*P*(*H*) / *P*(*D*). We use the form log(posterior odds) = log(prior odds) + log(likelihood ratio), where odds = *p*/(1 − *p*) and *p* = odds/(1 + odds); the prior odds begin at *P*(*H*) / *P*(¬*H*), the likelihood ratio (LR) is *P*(*D* | *H*) / *P*(*D* | ¬*H*), and the posterior odds are *P*(*H* | *D*) / *P*(¬*H* | *D*). Information from multiple identifiers, such as date of birth (DOB), forename, surname, etc. is thus combined using log likelihood ratios (LLRs): log(posterior odds) = log(prior odds) + LLR_DOB_ + LLR_forename_ + LLR_surname_ + … (etc.). Identifiers that are missing on either side contribute no information [9], known as the missing-at-random assumption [21].

A statistical assumption is that different identifiers are in general conditionally independent. For example, if everybody called Alice had the surname Smith, this assumption would be violated; the surname usually provides information, but would provide none for an Alice. This assumption is not always true; for example, forename frequency varies with year of birth. We make special provision for some cases, such as the non-independence of forenames and gender (see below).

### Approach to final selection among candidates

In this section we discuss how a winning candidate should be selected. If the sample and population are identical, meaning that the proband must be in the sample, it is easy to calculate accurately and choose a winner. Frequency information (e.g. the proportion of the population called Alice) could be calculated with certainty from the sample data. In a situation without data errors, a Bayesian calculation performed for each proband–candidate pair separately would give a final probability for each candidate, guaranteed to be accurate; those probabilities would sum to 1. A winner might not always be declared, because the information available may be insufficient: if the proband is called Alice, and the sample and population consist of two people named Alice, then both candidates have probability 0.5. More generally, choosing a “winner” requires setting a probability threshold.

Equally, if the sample were a random subset of the population, but the population frequencies were known exactly, this process would also be accurate.

However, inaccuracies in estimated population frequencies can give rise to inconsistent results, and this complicates the selection of a “winner”. Suppose the population and sample both contain two people named Alice (A1, A2) and 98 people named Bob, and the proband is named Alice. Consider the attempted match between the proband and A1. The probability of the proband being called Alice if they are not A1 is *P*(*D* | ¬*H*) = 1/99 ≈ 0.01, and *P*(proband is A1) = P(*H* | *D*) = 0.5.^1^ If we underestimate the frequency of the name Alice, however, such as by incorrectly estimating *P*(*D* | ¬*H*) = 0.001, then we will mistakenly calculate *P*(*H* | *D*) = 0.91, both for the proband–A1 comparison and for the proband–A2 comparison.^2^ In this mathematically incorrect situation, A1 looks by itself like an odds-on winner—and so does A2.

If the sample were a subset of the population, and population frequencies were potentially inaccurate, but the proband were guaranteed to be in the sample, then a normalization process would be possible to derive an absolute probability for the winner (Appendix 1; cf. [52]). However, in a realistic situation, many of these assumptions will not hold. The proband cannot be guaranteed to be in the sample. The sample is likely to be smaller than the population. Even if it was the entire population, practical considerations and IG rules may prevent transmission of sample frequency information to the provider of the probands, for the de-identified frequency encoding that we discuss later. The sample may be non-random with respect to the population: for example, a health service for under-18s will have DOBs distributed differently to the population as a whole. Frequency information may be imperfectly estimated.

We therefore adopt a simple heuristic two-stage process to selecting a winning candidate, which we show empirically to be robust despite the use of estimated population frequencies.

### Final selection method

Accordingly, we used the following approach. Having updated the odds according to each identifier (as set out below), two conditions are necessary to declare a match between a proband and a candidate. Firstly, the log odds of a match must exceed a threshold: the “consideration threshold”, *θ*. Secondly, the log odds of the leading candidate must exceed the log odds of the runner-up by another threshold: the “leader advantage threshold” or difference, *δ*, where *δ* ≥ 0 (Fig. 3C). Validation of this approach is shown below, including examination of good default values for *θ* and *δ*.

If *δ* = 0, *θ* behaves as the twin thresholds of the Fellegi–Sunter approach when they are set to be equal so as to give a binary partition between linked and unlinked pairs [9], except that Fellegi–Sunter classifies all proband–candidate pairs independently and thus can match a proband to multiple candidates, whereas our system selects at most one candidate for each proband, on the assumption that the sample contains distinct people. The additional option for a “leader advantage” *δ* offers the user a margin of certainty despite inaccuracies in prior probabilities, or when sparse information is insufficient to resolve very similar candidates. Requiring a “leader advantage” to declare a winner has obvious implications if the sample does contain duplicate records. If a person is present in the sample twice identically and *δ* > 0, they will never be selected, as copy 2 will be as good as copy 1 (no leader advantage). However, this does not prevent other people being matched successfully. If *δ* = 0, one will be selected; the system iterates through candidates in a consistent order and the first of any joint winners is preferred, thus also allowing the system to be used for potential duplicate detection if configured for self-linkage (e.g. by detecting events where > 1 proband matches to a single candidate) [10]. Rather than estimating thresholds based on desired error rates [9, 10], we report empirical performance by threshold values.

### Information not used

We do not use information about the non-equality of people within the sample, or of different probands. To illustrate this concept in the simplest case: if *n*_*s*_ = *n*_*p*_ = 2, then if the proband does not match sample member S1, and S1 ≠ S2, we are already certain that the proband is S2. Similarly, even if the sample is only a subset of the population, e.g. *n*_*s*_ = 100 < *n*_*p*_, then if we know that the proband did not match S1–S99, the probability that they match S100 must be somewhat higher than if we did not know the lack of match to S1–S99 (e.g. if *n*_*p*_ = 1000, then the probability that the proband is S100 has increased from ^1^/_1000_ to ^1^/_901_). A similar argument may be made from the non-equality of the probands (“if these other probands haven’t matched, the remainder are more likely”, or “this proband has been matched, so we can’t use its winning candidate for another proband”). We did not implement this kind of system, known as bipartite matching or one-to-one matching [10, 18, 20, 26, 53–55] for two reasons. One is computational: making every proband’s calculations dependent on all others is less efficient than comparing each proband separately to the sample. The other is that these assumptions may be too strong in practice: duplicate records occur.

### Error handling

Typographical and other data-entry errors make matching more difficult. An approach that accepts exact matches only will obviously fail to match when errors are present. Errors may also vary in degree and type (including, for example, single character changes; phonetically identical alternative name spellings; transposition of components such as day/month of birth, or first name/surname). With plaintext information, a continuous “distance” measure can be used to compare similar strings for typographical errors [25], but this approach is hard to discretize when creating a fully de-identified version (though see [34–36]). We adopted a simple generic approach compatible with de-identified matching, by defining several multi-level comparisons (see Table 1A–C, which defines the probability terms used below). For example, for some identifiers we used a three-state comparison involving a full match, a partial match, or no match.

**Table 1:** Systems for full and partial matching, applicable to different identifier types. *D*, data; *H*, hypothesis that the proband and candidate under consideration are the same person. For each system, columns sum to 1, since the options for *D* are mutually exclusive. (A) Two-state system: a match occurs or does not occur. There is a probability* p*_*c*_ (c, correct) that an identifier is correctly represented (is the same) when the proband and candidate are the same person, and a probability *p*_*e*_ = *p*_*en*_ (e, any error; en, error yielding no match) that an error or mismatch occurs. In the population, there is a probability* p*_*f*_ (f, full match) that a randomly selected other person shares the proband’s identifier, and a probability *p*_*n*_ (n, no match) that they do not. (B) Three-state (“fuzzy”) system. The nature of a partial match is specific to the identifier type. For example, for date of birth (DOB), the partial match is a DOB with 2/3 of year/month/day correct. Probabilities* p*_*c*_,* p*_*f*_,* p*_*en*_, and *p*_*n*_ are as before, but now there is a probability* p*_*ep*_ (ep, error yielding partial match) that when the proband and candidate are the same person, the identifiers match only partially (so *p*_*e*_ = *p*_*ep*_ + *p*_*en*_), and a probability *p*_*pnf*_ that that a random other person will share the partial but not the full identifier. It may be easier to measure *p*_*p*_, the total probability of a partial or full match, than *p*_*pnf*_. (C) Four-state system. Partial matches now occur in two variants, hierarchically. (D) Adjustments for unordered pick-the-best comparisons between multiple identifiers of the same type (e.g. surnames, postcodes). “Positive” comparisons are those for which the log likelihood ratio is > 0. (E) Adjustments for ordered pick-the-best comparisons between multiple identifiers of the same type (e.g. forenames), using the probability *p*_*o*_ (o, ordered) that, given *H*, for ≥ 2 candidate identifiers, the candidate’s order strictly matches the proband’s, and its converse probability *p*_*u*_ (u, unordered). Positive comparisons are strictly ordered when each proband identifier’s index matches the corresponding candidate’s identifier. † For *P*(*D* | ¬*H*), adjustments use the Bonferroni correction (see text)

Data, *D*	*P*(*D* | *H*, same person)	*P*(*D* | ¬*H*, different person)
**A. Two-state comparison**		
Match	*p*_*c*_ = 1 − *p*_*e*_ = 1 − *p*_*en*_	*p*_*f*_
No match	*p*_*e*_ = *p*_*en*_	*p*_*n*_ = 1 − *p*_*f*_
**B. Three-state comparison**		
Full match	*p*_*c*_ = 1 − *p*_*e*_ = 1 − *p*_*ep*_ − *p*_*en*_	*p*_*f*_
Partial (but not full) match	*p*_*ep*_	*p*_*pnf*_ = *p*_*p*_ − *p*_*f*_
No match	*p*_*en*_	*p*_*n*_ = 1 − *p*_*p*_
**C. Four-state comparison**		
Full match	*p*_*c*_ = 1 − *p*_*e*_ = 1 − *p*_*ep1*_ − *p*_*ep2np1*_ − *p*_*en*_	*p*_*f*_
Partial match type 1 (but not full)	*p*_*ep1*_	*p*_*p1nf*_ = *p*_*p1*_ − *p*_*f*_
Partial match type 2 (but not full or partial type 1)	*p*_*ep2np1*_	*p*_*p2np1*_
No match	*p*_*en*_	*p*_*n*_ = 1 − *p*_*p*_ = 1 − *p*_*p2np1*_ − *p*_*p2nf*_
**D. Adjustments for unordered multi-identifier comparison**		
For 1 ≤ *c* ≤ min(*n*, *m*) “positive” comparisons between proband identifiers 1…*n* and candidate identifiers 1…*m* †	× 1, no correction	$\times \prod^{c-1}_{i=0} \left(m-i\right)$
**E. Adjustments for ordered multi-identifier comparison**		
For *c* ≥ 1 “positive” comparisons, *m* > 1, and strict order match	× *p*_*o*_	× 1, no correction
For *c* ≥ 1 “positive” comparisons, *m* > 1, and order mismatch †	× *p*_*u*_ = 1 − *p*_*o*_	$\times \left(\left[\prod^{c-1}_{i=0} \left(m-i\right)\right]-1\right)$

### Corrections for multiple comparisons

In some situations, a proband and/or candidate may have multiple identifiers of a class, e.g. forename or postcode. The order in a given database may be irrelevant (e.g. postcode) or relevant (e.g. forename). Comparing multiple pairs of identifiers and selecting the best match or matches increases the probability that a random candidate will match by chance, so we corrected for multiple comparisons as follows.

#### Unordered comparisons

For a proband with *n* identifiers and a candidate with *m* identifiers, we compared all proband–candidate pairs, and sorted them by log likelihood ratio (LLR). We took LLR > 0 to indicate a “match” of some sort. Each identifier was only permitted to contribute once, so there can be *c* ≤ min(*n*, *m*) successful “match” comparisons. For the illustrations below, we suppose the population of all identifiers is {A, B, …, Z}, giving a set of size *s* = 26, and that every identifier is equiprobable in the population with frequency *q* = 1/*s* = 1/26.

*P*(*D* | *H*) does not require correction in this situation: if the proband is [A, B], whether the candidate is [A, B] or [B, A] does not matter: both would be treated as giving A–A and B–B matches.

However, *P*(D | ¬*H*) may require correction in some circumstances. Corrections are not required if multiple comparisons are not in fact made. If the proband has a single identifier [A] and the candidate also has a single identifier, then we will declare a match if the candidate is A, an event whose probability for a random candidate is *P*(*D* | ¬*H*) = *q* = 1/26, and this situation requires no further correction.

If the proband is [A] and our candidate has two identifiers (*n* = 1, *m* = 2), a match will be declared when the candidate is [A, ?] or [?, A]. We would therefore declare a match with a random candidate [?, ?] with probability 1/26 + 1/26 − 1/26^2^ = 2*q* − *q*^2^. The subtracted component is for a candidate [A, A], who would otherwise be counted twice. Generalizing, for* n* = 1, the probability of a random candidate matching is 1 − (1 − *q*)^*m*^, because the non-match probability for each candidate identifier is (1 − *q*) and it takes *m* individual non-matches to achieve an overall failure to match. By the Bonferroni approximation, this is approximately *mq*, and never more, so *mq* is a slightly conservative correction for multiple comparisons. We used the Bonferroni correction rather than the accurate value because this is substantially easier to implement in a cumulative log odds system with varying *q*. Since an uncorrected comparison would give *P*(*D* | ¬*H*) = *q*, the correction can be implemented by adding − ln(*m*) to the cumulative log odds.

Generalizing further to multi-named probands, we can work sequentially: the first proband name is matched by the candidate with approximate probability *mq*_1_; then, having used up one candidate name, the second proband name is matched by the candidate with approximate probability (*m* − 1)*q*_2_, and so on. We implement this by adding − ln(*m*⋅[*m* − 1]⋅…) to the cumulative log odds.

No correction is required for “non-match” comparisons, however, since no “fishing” for the correct order is then needed. This process is summarized in Table 1D.

#### Ordered comparisons

If the order of the comparisons is significant (e.g. forenames), we modify the process slightly. If only one comparison could be made, no correction was necessary. Otherwise we proceed as follows.

For *P*(*D* | *H*), we define *p*_*o*_ as the probability of an ordered match (given *H*) if there is more than one possible comparison ordering, and its converse *p*_*u*_ = 1 − *p*_*o*_, the probability of an unordered match in these circumstances, which can be thought of as the probability of a recording error that alters the sequencing of these identifiers. We ranked potential pairwise matches and picked the most likely as before. If that best set of matches reflected strict ordering, such that the proband’s and candidate’s identifier indices were identical for all pairs (e.g. P1–C1 and P2–C2), we weighted *P*(*D* | *H*) by *p*_*o*_, by adding ln(*p*_*o*_) to the cumulative log odds. There was only one way in which an ordered match could be achieved. Otherwise, if any matches occurred (defined as LLR > 0 per comparison), we weighted *P*(*D* | *H*) by *p*_*u*_.

For *P*(*D* | ¬*H*), we did not apply a correction if the matches were strictly ordered, since there is only one way in which such a combination can be achieved. If there was a set of matches that were not strictly ordered, we applied the same correction as for the unordered process (as above), subtracting one (for the strictly ordered option). This process is summarized in Table 1E.

### Linkage using direct identifiers

We applied this system to the following types of identifier.

#### Date of birth (DOB)

DOB is a core personal identifier that always exists (in principle) and should never change. We used a three-state comparison (Table 1). A full match was an exactly equal DOB. Based on empirical data (see below), we defined a partial category as being a DOB that differs in a single category only—year, month, or day. Any other DOB was considered a mismatch. We presume that the population frequency *p*_*f*_^*DOB*^ = *P*(same DOB | different person) = ^1^/_365.25⋅*b*_ where *b* is a configurable parameter related to the birth year range—the age range but including deceased people—of the population of interest. The parameter* b* reflects the intuitive concept that if everyone in the population of interest is aged 42, the chance of an exact DOB (year–month–day) match between two random people is about ^1^/_365_, whereas in a population with an evenly distributed age range of 1–100, it is about ^1^/_36500_. In populations with an uneven DOB distribution, *b* may be lower than the full range (an intuitive example: one person is aged 1, one person is aged 100, and everyone else is aged 42). In principle, a birthday of 29 February is ~4 times less common than all others, so it may merit special treatment, but for simplicity, and to reduce re-identification risks from marking it as different in de-identified data, we treated it just like all other days. We calculated *p*_*pnf*_^*DOB*^ and *p*_*n*_^*DOB*^ accordingly, using the birth year range, the number of months per year, and the mean number of days per month (*p*_*pnf*_^*DOB*^ = ^1^/_365.25_ + ^1^/_30.4375⋅*b*_ + ^1^/_12⋅*b*_ − ^3^/_365.25⋅*b*_ = ^(16⋅*b* + 631)^/_5844⋅*b*_). We took error rates *p*_*ep*_^*DOB*^ and *p*_*en*_^*DOB*^ as configurable parameters.

#### Gender

We used standard genders of F (female), M (male), X (other) [56]. Given three possibilities only, we used a two-state comparison (Table 1) with no partial match option. We took *p*_*en*_^*gender*^ = *P*(different gender | same person), and *p*_*f*_^*gender*^ = *P*(same gender | different person), which depends on the gender in question, as configurable parameters. Not all systems may allow three-state recording and the UK Census did not ask about gender in addition to sex prior to 2021. If gender was absent from the proband or candidate, we did not infer evidence.

#### Forenames

A forename is any name that precedes a surname (i.e. first names and middle names; given names). When comparing an individual name between proband and candidate, we used a four-state comparison (Table 1). Exact matches were for names, standardized to remove punctuation and whitespace, converted to upper case, and with accents removed (e.g. Ü → U). Primary (preferred) partial matches were for the name’s metaphone, specifically the first part of the result of the double metaphone algorithm [57]. This is an approximate phonetic algorithm that allows for some kinds of typographical error by mapping, for example, {Rudolf, Rudolph} → RTLF; {Jonathan, Jonathon} → JN0N. Secondary partial matches were for the first two characters (F2C) of the name.

Gender and forename are not statistically independent. Forenames vary from those that are very strongly predictive of gender (in English-speaking countries e.g. Elizabeth, John) to those with relatively weak gender associations (e.g. Rowan); the gender association also varies with country of birth (e.g. Andrea). Therefore, we made all forename-related frequencies conditional upon the proband’s gender: for example, for a female proband, the population frequency was that among females.

We calculated population frequencies for names, metaphones, and F2C from public US historical baby name frequencies [58]. For a proband of unknown or X gender, we used the weighted mean of F/M frequencies (the whole-population frequency). For any names that did not have metaphones, we could still calculate a population frequency for “null” metaphones. For names unknown to the large public data set, we used a user-configurable standard minimum frequency. We used gender-specific configurable parameters for error rates, with defaults based on empirical data (see Table 2 and below).

**Table 2:** Settings used for the validation experiment. These settings are all configurable by the user. F2C, first two characters. § Values encoded directly or indirectly in the proband file for hashed comparisons; other values set at comparison time. ¶ For probands with gender X or absent gender, the weighted mean of F/M values was used. † From empirical data from a single database or database pair in this study; see Results

Setting	Value	Comment
**§ SECURITY**		
Hash method	HMAC-MD5	HMAC-MD5 has a space of 16^32^ = 3.40 × 10^38^. The software also offers HMAC-SHA-256 (space size 16^64^ = 1.16 × 10^77^) and HMAC-SHA-512 (space size 16^128^ = 1.34 × 10^154^)
Number of significant figures for rounding frequencies in hashed version	5	Rounding reduces the identifiability of numbers. Some precision is required to distinguish metaphone from name frequencies
**§ POPULATION PRIORS: NATIONAL**		
Name/metaphone/F2C frequencies for forenames, by gender	[many]	¶ From US baby name frequencies 1880–2015 [58], covering ~345 M people, processed via CRATE [59]. UK data by year is also available [60]
Name/metaphone/F2C frequencies for surnames	[many]	From US 1990 and 2010 Census surname frequencies [61, 62], processed via CRATE [59]
Minimum frequency for forenames, *f*_*min*_^*forename*^. (If a frequency was less than this, this minimum was used instead.)	5 × 10^−6^	A minimum is required for unknown names. For the US forename data cited, the floor frequency is ~2.9 × 10^−8^; however, allowing extremely low frequencies (e.g. much below 1/*n*_*p*_) increases the chances of a spurious match, because a name match can add up to ln(1/*f*_*min*_) to the log odds
Minimum frequency for surnames, *f*_*min*_^*surname*^	5 × 10^−6^	As above. For the US surname data cited, the lowest frequency reported is 3 × 10^−7^, but we used a threshold above 1/*n*_*p*_
* P*(female | female or male)	0.51	With a binary sex choice, the UK is 51% female and 49% male [63]
* P*(not female or male)	0.004	Approximately 0.4% of the UK consider their gender neither male nor female [64]
Postcode data, for *p*_*f*_^*postcode*^, *p*_*pnf*_^*postcode*^, and *p*_*n*_^*postcode*^	–	From UK Office for National Statistics data [65], licensed under the Open Government Licence version 3.0
**POPULATION PRIORS: LOCAL**		
Population size, *n*_*p*_	852,523	Population estimate of Cambridgeshire and Peterborough for 2018 [66]
Birth year “range” *b*	30	† The prior probability of two people sharing a DOB was taken as ^1^/_365.25⋅*b*_. A value of 90 may be reasonable for a full UK population with few long-deceased people [67], but we used an empirical value reflecting the subsampled age composition of one of our databases
Postcode frequency multiple *k*_*postcode*_	*n*_*UK*_/*n*_*p*_	Where *n*_*UK*_ is the 2017 UK population, 66,040,000 [67]
Population proportion assumed to be assigned a pseudopostcode (e.g. ZZ99 3VZ, no fixed abode; ZZ99 3CZ, England/UK not otherwise specified) or a postcode unknown to the postcode database (including typographical errors creating an invalid postcode), *p*_*pseudopostcode_unit*_. Taken as an estimate for each unknown/pseudopostcode unit frequency	0.00201	† Based on the proportion of people in the SystmOne database with a ZZ99 3VZ (no fixed abode) postcode. This is higher than an estimate from national data (see Results), potentially reflecting a bias from a healthcare environment, so this value may need alteration in other contexts
Pseudopostcode multiple *k*_*pseudopostcode*_ such that *p*_*pseudopostcode_sector*_ = *k*_*pseudopostcode*_ × *p*_*pseudopostcode_unit*_	1.83	† Based on an empirical value for ZZ993:ZZ993VZ (see Results). This number cannot be < 1 and should be > 1 to avoid *p*_*pnf*_^*postcode*^ = 0
**ERROR RATES (given proband/candidate are the same person)**		
* p*_*ep1*_^*forename*^	F: 0.00894 M: 0.00840	†¶ Probability that a forename pair exhibits partial 1 (metaphone) match but not a full (name) match
* p*_*ep2np1*_^*forename*^	F: 0.00881 M: 0.00688	†¶ Probability that a forename pair exhibits a partial 2 (F2C) match, but not a partial 1 (metaphone) or full (name) match
* p*_*en*_^*forename*^	F: 0.00572 M: 0.00625	†¶ Probability that a forename pair exhibits no match at all
* p*_*u*_^*forename*^	0.00191	† Probability, amongst a set of ≥ 2 forenames, of an error that shuffles the names out of strict order
* p*_*ep1*_^*surname*^	F: 0.00551 M: 0.00471	†¶ Probability that a surname pair exhibits a partial 1 (metaphone) match but not a full (name) match
* p*_*ep2np1*_^*surname*^	F: 0.00378 M: 0.00247	†¶ Probability that a surname pair exhibits a partial 2 (F2C) match, but not a partial 1 (metaphone) or full (name) match
* p*_*en*_^*surname*^	F: 0.0567 M: 0.0134	†¶ Probability that a surname pair exhibits no match at all
* p*_*ep*_^*dob*^	0.00459	† Probability of a DOB error causing a partial (year/month, month/day, or year/day) match
* p*_*en*_^*dob*^	0	The probability of a DOB error causing no match at all. Using 0 rather than the empirical value of 0.00033 produces a major speed advantage; see Results
* p*_*e*_^*gender*^	0.0033	† The probability that proband/candidate (when the same person) do not match on gender
* p*_*ep*_^*postcode*^	0.0097	† The probability that a proband/candidate postcode pair (when the same person) exhibits a partial (postcode sector) match but not a full (postcode unit) match, e.g. due to error or because someone has moved within a postcode sector
* p*_*en*_^*postcode*^	0.300	† The probability that two postcodes for the same person mismatch completely

If applicable, we applied a correction for ordered multi-identifier comparisons (Table 1E), using *p*_*o*_^*forename*^ and *p*_*u*_^*forename*^.

In this system, if either person had no forename recorded, no evidence was inferred. Optionally, we allowed start/end dates to be associated with any forename. If there was an explicit temporal non-overlap between any two names, they were not compared.

#### Surname

We used broadly the same approach for surnames (last names, family names) as for forenames, with two modifications.

First, surnames may contain multiple components (e.g. Smith-Jones, van Beethoven), which may be recorded variably. To handle this problem, we split each surname into ≥ 1 fragments, by splitting whitespace- or punctuation-separated parts of double-barrelled surnames and standardizing the whole component by removing whitespace/punctuation (e.g. L’Estrange → LESTRANGE; Mozart-Smith → MOZARTSMITH, MOZART, SMITH), and by creating versions with accents removed and transliterated (e.g. Müller → MÜLLER, MULLER, MUELLER). We maintained a user-configurable list of nonspecific name components to ignore, such as nobiliary particles (thus: van Beethoven → VANBEETHOVEN, BEETHOVEN, but not VAN). Individual fragments were compared using the four-state method described as above. For a given name comparison, when choosing which fragments to compare, we preferred full match > metaphone match > F2C match > no match, and within each category preferred the most informative match (thus, for example, proband “Mozart-Smith” versus candidate “Mozart-Smith” would yield a match on MOZARTSMITH, not SMITH). Population frequencies were taken from the fragment that did in fact match; thus, for example, for proband “Mozart-Smith” versus candidate “Smith”, the population frequency was taken to be that of the fragment SMITH. We used a large-scale US dataset for surname frequencies [61, 62], described below, which also uses a “no space, no punctuation” standardization.

Second, we regarded multiple recorded surnames (if present) as representing alternatives, not sequenced names, so we used an unordered multiple-comparisons method (Table 1D). We did not allow for forename/surname transposition.

#### Postcode

This part of the system is specific to the UK, but adaptable in principle to similar systems internationally. UK addresses all have a postcode, which is a 5–7-character alphanumeric string. Postcodes have four nested components: area (the largest geographical size, e.g. a city and its surroundings), district, sector, and unit (the smallest geographical size, typically a street or part of a street). Postcodes are a relatively weak identifier because people move home, so a postcode mismatch between two databases is not uncommon, but sharing a postcode at a given point in time provides some evidence towards an identity match. We allowed postcodes to be associated with a start date and an end date, though these can be null, e.g. for an unknown start date or an absent end date representing a current postcode. We used a three-state comparison (Table 1), in which the partial match was a match on postcode sector (i.e. same area, district, and sector, but not unit). We compared postcodes in unordered fashion (Table 1D), ignoring postcode pairs that explicitly did not overlap in time. Differing postcodes were used as evidence against a match, but this was weak evidence given the high prior probability of non-matching, *p*_*en*_^*postcode*^. We did not use information about the duration of the period for which a postcode is shared by the proband and sample except to exclude matches when the overlap was zero, as above.

For population priors, we first calculated national frequencies *f*_*f*_^*postcode*^ = postcode population ÷ total UK population, and *f*_*p*_^*postcode*^ = sector population ÷ total UK population. Postcode and sector populations were estimated from national data. Postcodes contribute to larger units called Output Areas (OA) [68], OAs combine to form sectors, and the nationwide mean OA population is published. We estimated the population for each postcode, as the mean OA population divided by the number of postcodes in a given postcode’s OA [65]. We estimated postcode sector population as the mean OA population multiplied by the number of OAs in a given sector. We then allowed some flexibility in calculating postcode probabilities, by allowing users to specify a probability multiple *k*_*postcode*_, such that *p*_*f*_^*postcode*^ = *k*_*postcode*_*⋅f*_*f*_^*postcode*^ and *p*_*p*_^*postcode*^ = *P*(same sector | different person) = *k*_*postcode*_*⋅f*_*p*_^*postcode*^. This is because users may have local populations that are geographically restricted, e.g. the population under consideration might represent 1% of the national population coming from only 1% of the national postcodes (*k*_*postcode*_ ≈ *n*_*UK*_/*n*_*p*_), or evenly distributed, e.g. 1% of the national population distributed across national postcodes (*k*_*postcode*_ ≈ 1). We determined error priors empirically (see below).

In the UK, some people are assigned a “pseudopostcode”: for example, ZZ99 3VZ indicates “no fixed abode” [69, 70]. The recording of such a postcode is informative, and homelessness represents a potential barrier to linkage, so we did not ignore these. We assigned a parameter representing the probability *p*_*pseudopostcode_unit*_ that a random person would have a postcode unit recognized as a pseudopostcode (or a postcode unit unknown to the national postcode database, including typographical errors). It is highly desirable to have the corresponding sector probability greater than the unit probability (to be less is nonsensical and if they are equal then *p*_*pnf*_^*postcode*^ = 0 so an erroneous partial match gives LLR = ∞), so we specified *p*_*pseudopostcode_sector*_ = *k*_*pseudopostcode*_*⋅p*_*pseudopostcode_unit*_, where *k*_*pseudopostcode*_ is configurable. We weighted all other postcode frequencies by (1 − *p*_*pseudopostcode_sector*_).

### Within-comparison ordering checks

User-configurable parameters can alter the ordering of possibilities within comparisons. For a three-state comparison, one would intuitively expect this LLR ordering: no match ≤ partial match ≤ full match. However, this is not an absolute requirement. For example, with standard settings, the male forename JAMES has *p*_*f*_ = 0.0295 and *p*_*p1nf*_ = 0.000133 (“metaphone JMS but forename not JAMES”), i.e. other names are very unlikely to generate this metaphone. With *p*_*c*_ = 0.978 and *p*_*ep1*_ = 0.0084, a metaphone-not-full match (LLR +4.15, e.g. JAMES–JAIMES) is slightly better evidence for *H* than a full match (LLR +3.50). Even the “no-match” comparison is not guaranteed to be the minimum. For example, surname ALLEN has *p*_*p2np1*_ = 0.110 (“starts AL but not ALLEN”) and *p*_*n*_ = 0.887, i.e. many other names share this F2C. With *p*_*ep2np1*_ = 0.00314 and *p*_*en*_ = 0.0355, a F2C-not-metaphone-or-name match (LLR −3.56, e.g. ALLEN–ALLARDYCE) provides slightly worse evidence for *H* than a complete mismatch (LLR −3.22). In our implementation, the user has the option to check all comparisons and see warnings if the order deviates from none ≤ partial(s) ≤ full.

### Linkage without direct identifiers

We now proceed to eliminate the need for direct identifiers (Fig. 3B).

#### Cryptographic hash function

The core cryptographic technique used is that of a secure hash [71, 72]. A hash function “boils down” complex input data of arbitrary size to a simple, fixed-size value or “digest”. Good hash functions do this in a way that any two inputs are extremely unlikely to produce the same digest: “collisions” are few. Thus, if two digests from the same hash function are identical, it is extremely likely that the inputs are identical—we assume that this is certain. Cryptographic hash functions are infeasible to invert. They are typically set up using a secret hash “key”, such as a phrase or complex character sequence. For a given key, one can hash input data, consistently but irreversibly, to unique output values—but a change in the key will change the digest. Therefore, given a shared secret key, organisations A and B can hash their identifiers to non-identifying values that can be compared. The agent performing the linkage does not need the key to compare the digests. (Note that the secrecy of the key should be treated with some respect. Suppose an organization uses its key to hash NHS numbers. It is computationally infeasible to calculate an NHS number from a digest—but there are only about 10^9^ NHS numbers, so if a malicious agent discovers the key and the nature of the hash function, it would be trivial to compute the digests of all possible NHS numbers for that key, and thus to infer an NHS number from a digest if the attacker also had access to that information.)

#### De-identified linkage using a hashed common unique identifier

If A and B both use a common unique identifier (such as an NHS number), it is trivial to match identities without direct (plaintext) identifiers being shared. A and B share a secret hash key, generated and reserved for this purpose. Separately, A and B both prepare de-identified versions of their data in which the common identifier is hashed using the shared key to produce a “research ID” that is not itself directly identifying. The de-identified data, with people tagged by their research ID, can then be shared securely and linked on the research ID (e.g. Figure 2B,D).

#### De-identified linkage without a common unique identifier

De-identified linkage without a common unique identifier is more complex, particularly in the presence of potential errors, but now follows directly from our Bayesian method. Using the agreed key, organizations A and B prepare a data file of their relevant patients (e.g. probands from A to be matched to B’s sample). Direct identifiers are hashed, yielding digests that can be compared for exact matches. For Bayesian and “fuzzy” matching, while it is possible to transform a plaintext first name into a metaphone at any time for fuzzy comparison, or establish its population frequency for Bayesian calculation, this is not possible with digests. Therefore, in our approach, the originating organization hashes the identifier and associated fuzzy (imprecise) version, and provides corresponding frequency information too (e.g. hashed name, name frequency, hashed name metaphone, and metaphone frequency), allowing for the same Bayesian linkage process as with plaintext.

This approach hashes tailored fuzzy information for each relevant identifier. There are other algorithms for fuzzy hashing, but we note the unsuitability of many. Most are designed for long streams of bytes or text, and in general, they chop the input up into blocks or overlapping blocks, hash each block, and then compare the sequence of mini-hashes for similarity [73, 74].

#### Providing frequency information:proband or candidate?

If “complete” errors are taken to prohibit a match for a given comparison,* p*_*en*_ = *P*(complete mismatch | *H*) = 0, frequency information might be provided with either the proband or the candidate. Which should we use? Suppose we applied this mechanism to forenames, that the proband is Alice, and the population has 6 × Alice and 4 × Bob. When the candidate is Alice, this is a match, and *p*_*f*_ relates to the population frequency of “Alice”,^3^ identifiable from information associated with either the proband or the candidate. Likewise, for a partial match via the corresponding metaphone ALK, frequency information is available via the proband or the candidate. When the candidate is Bob, there is no match, so *P*(*D* | *H*) = 0 and the LR is 0, making *P*(*D* | ¬*H*) irrelevant.^4^ We eliminate Bob without having to know the probability of an “Alice–other” mismatch, *P*(not Alice and not ALK | ¬*H*).

In contrast, for comparisons where “complete” errors do not completely prohibit a match, such as gender (see above), the direction of comparison matters. If the proband Alice has gender F and the candidate Bob has gender X, then *P*(*D* | ¬*H*) = *P*(randomly selected different person does not have the *proband’s* gender). Since gender has a restricted known set of possibilities, special measures would be possible to enable frequency information to be stored only with the sample (inferring inverse frequencies from frequencies associated with hashed genders in the sample, or hashing them afresh from user-supplied information). However, the principle that “frequency information is provided with the proband” allows the system to be extended such that *P*(complete mismatch | *H*) > 0 for other identifiers, where the process might not be as simple as for gender. For example, knowing the frequency of “not hashed Alice” would be impossible from sample information if “hashed Alice” is not even present in the sample.

Because the relevant frequencies are associated with the probands, in our implementation they must be present in the proband file, but are not required in the sample file. The assumption is that the population frequencies known to the proband sender match those applicable to the sample. Additionally, a small amount of person-independent information is supplied at comparison time, including for the baseline log odds and for postcode frequency adjustments; this allows a single proband file to be used more readily for multiple comparisons with different organizations. Table 2 shows what is encoded in the hashed proband file and what is supplied at comparison time.

### Limitations

Some groups may be more vulnerable to particular categories of error. For example, typing errors may be commoner in names that are rarer in a given country; some cultures have names with multiple possible transliterations into a Latin alphabet; first name/surname transposition may be commoner when Chinese names are transcribed in Western countries (Chinese names are written and spoken as *Familyname Givenname*, sometimes anglicized as *Givenname Familyname*); some people use a middle name for preference; gender/sex errors may be commoner for people who are transgender. Some but not all of these are likely to be addressed by our use of “sounds-like” partial matches for names, our forename sequence handling, and a degree of automatic transliteration handling. Our general approach, however, is extensible to other types of error.

### Implementation

We implemented the linkage system in Python as part of the free and open-source package CRATE (Clinical Records Anonymisation and Text Extraction) [75]. We offer HMAC-MD5, HMAC-SHA-256, and HMAC-SHA-512 as cryptographic hash functions [75] (HMAC, hash-based message authentication code; MD, message digest; SHA, Secure Hash Algorithm). Metaphones were calculated via the “Fuzzy” package [76].

Considering each proband separately makes the linkage of a large number of probands an “embarrassingly parallel” problem (parallelizing over probands) and the software supports parallel processing via a multi-process model, though performance varies with operating system (e.g. performance is worse under Windows than Linux due to differences in process startup methodology) and the overhead associated with process launching means that parallel processing may not be worthwhile for small data sets.

If the user sets *p*_*en*_^*DOB*^ = 0, candidates are pre-filtered to those sharing an exact or partial DOB match, as this is considerably more efficient (with evenly distributed birthdays, by a factor of 5844⋅*b* / [16⋅*b* + 647], e.g. for *b* = 100, ~260 times faster), with a further speedup if *p*_*ep*_^*DOB*^ = 0 also (a further *b* + 647 / 16 ≈ 140 times faster in this example, for a total speedup of 365.25⋅*b*). In addition, of necessity, probands with no DOB are always compared to all candidates, and all candidates with no DOB are always compared to each proband.

In our basic implementation, a file for plaintext linkage is a comma-separated value (CSV) or JavaScript Object Notation Lines (JSONL) [77] file with one row per person, containing:

• a database-unique identifier (local ID, e.g. organization A’s unique identifier, or B’s), not used for linkage but to allow “read-out” of linkage;
• forename(s) (*);
• surname(s) (*);
• DOB;
• gender;
• postcode(s) (*);
• optional named “perfect” (person-unique) identifiers (e.g. NHS number, National Insurance/social security number), if the user wishes to use a blend of deterministic and probabilistic matching;
• optional arbitrary user-defined information (e.g. for users to conduct their own validation by associating gold-standard linkage information with each record).

Identifiers marked (*) can have optional start/end dates. The corresponding file for de-identified linkage is in JSONL format and provides information about:

• database-unique identifier (local ID, for linkage read-out);
• hashed forenames, with their hashed metaphones and hashed F2C, and corresponding frequencies (*);
• hashed surnames, similarly (*);
• hashed DOB;
• hashed gender, gender frequency;
• hashed postcodes, with their hashed sectors, and corresponding frequencies (*);
• hashed “perfect” identifiers, if desired;
• optional user-defined information (unmodified), if desired, e.g. for validation.

Identifiers marked (*) can have optional plaintext start/end dates. Frequencies are required in the proband file but not the sample file, and are rounded to a user-defined number of significant figures to reduce identifiability further. Hashing gender adds little in terms of information removal but prevents any plaintext identifiers from appearing to casual observation. The file for hashed linkage is automatically generated from the equivalent plaintext file. The local ID can be hashed using a separate key, to avoid sharing this key with the other organization, or left unmodified (e.g. if pre-hashed using an alternative method). If required, optional additional information can be attached to each row and preserved through linkage (e.g. for the present validation study).

Two de-identified (or identifiable) files can be compared automatically. For multi-way linkage, users might wish to build a single file of people and use it for fuzzy linkage with some organizations and exact linkage with others, or a blend, so “perfect” person-unique identifiers can also be used for matching. A match on any “perfect” identifier will yield that candidate only. The software allows translation of the names of these perfect identifiers to equivalents, e.g. if one organization builds its data file referring to “nhsnum” and another to “nhs_number”.

## Validation

### Methods

#### Source data

We took advantage of a natural experiment within CPFT, in which four multiple clinical record systems had both overlapping and distinct patients but shared a common person-unique identifier type (a UK NHS number). These databases were created separately and had separate patient registration processes, making them useful for comparison. They were:

- 1. CDL. An early in-house mental health (MH) clinical records system, CRS/CDL (Care Records System/Clinical Document Library; “CDL” for short), was most active from 2005–2012.
- 2. RiO. CDL was replaced in 2013 by another electronic records system, Servelec’s RiO. When RiO was launched, patients with active referrals, or who had been referred within the preceding 6 months, were migrated (by humans) from one system to the other. Thus, some patients (e.g. referred long before 2013 and never since) have records in CDL but not RiO. Others (referred for the first time after RiO launched) have records in RiO but not CDL. Yet others are present in both systems—including those who were migrated in the transition, but also those who have been re-registered since (e.g. referred in 2010, referral closed in 2010, not migrated to RiO at its launch, re-referred in 2015). Though there is also some checking via the national NHS Spine database of NHS number registrations, manual and independent data entry creates the potential for error (e.g. typographical errors in names), and people’s names can change, as can their gender and their address. The NHS number represents a gold standard of linkage, and contains a checksum to prevent simple typographical errors. Two people from the two databases who shared an NHS number were considered the same person.
- 3. SystmOne. A similar process took place when RiO was replaced by SystmOne (from The Phoenix Partnership, TPP) for MH data in three phases during 2020–21. SystmOne had also previously been in use for community physical health services, which moved from a different Trust to CPFT in 2015. It has real-time NHS Spine connectivity.
- 4. PCMIS. Finally, CPFT uses PCMIS (Patient Case Management Information System) software (originally developed at the University of York) for its Improving Access to Psychological Therapy (IAPT) service.

These databases also vary in terms of their coding, e.g. of middle names and historical postcodes. More details are reported below.

#### Approvals

These identifiable databases are routinely de-identified into the CPFT Research Database (NHS Research Ethics references 12/EE/0407, 17/EE/0442, 22/EE/0264), under the supervision of technical administrative staff with the authority to handle original identifiable NHS data. The current study was approved by the CPFT Research Database Oversight Committee. Data were extracted and fed through an automatic de-identification and linkage pipeline before analysis.

#### Patient and public involvement

Patient and public involvement (PPI) representatives of the CPFT Research Database Oversight Committee were consulted about the project in advance and endorsed its purpose and methods.

#### Computing environment, benchmarking, and analysis

The work was conducted on a multi-user Hewlett Packard Enterprise ProLiant DL360 Gen9 computer with two Intel Xeon E5-2687W v4 3 GHz processors, 672 Gb RAM, and solid-state disk storage, running Windows Server 2012 R2 (version 6.3), within a CPFT secure computing environment.

We measured the time to create a hashed identity file from the identifiable file, and the time to perform de-identified linkage (excluding the time to load the hashed identity files). To perform linkage, the software loads proband/candidate data, opens a results file, performs the comparisons, and closes the results file. We timed from opening to closing the results file, and benchmarked database self-comparisons using 24 logical processors; this was slower than single-process mode for small comparisons but became substantially faster for larger comparisons.

Analyses were performed in R version 4.0.3 [78]. We used Type III sums of squares for analysis of variance, and *α* = 0.05. For receiver operating characteristic calculations, we used the pROC package [79] with calculated log odds as the predictor (replacing −∞ with the arbitrary low finite value −10^5^ for this purpose); the response variable was whether the proband was in the sample (see also below and Appendix 2).

#### Population frequencies

As described above, population frequencies are used by the algorithm for *P*(*D* | ¬*H*). To establish probabilities such as the likelihood of two randomly selected people sharing a surname metaphone but not a name (etc.), we iterated through name/frequency pairs in the public name databases calculating Σ_*A*_Σ_*B*_* p*_*a*_*p*_*b*_*x*, where *A* and *B* are identical copies of the name set, *x* ∈ {0, 1} is a binary variable representing occurrence of the event in question for the combination of names *a* ∈ *A* and *b* ∈ *B* (e.g. metaphone match but not name match), and *p*_*a*_ and *p*_*b*_ are the population frequencies (probabilities) of the name in question. We normalized to Σ_*A*_*p*_*a*_ = Σ_*B*_*p*_*b*_ = 1.

We obtained empirical measure of *p*_*f*_^*DOB*^ and *p*_*pnf*_^*DOB*^, and thus *b*, by linking (in the SystmOne database) a small subsample of people, chosen arbitrarily by the first two digits of NHS number, and linked to all others in the subsample excluding themselves (~19 × 10^3^ people, ~3.6 × 10^8^ pairs). (In a true population one would include self-linkages but in this small sample that over-enriched for DOB matches, empirically by ~1.6-fold over disallowing self-matches.) This process treats this database as an unbiased estimator of the population (thus the largest database was used), and is unlikely to privilege linkage involving this database; however, all other database pairings represent checks of linkage accuracy that eliminate this possibility entirely.

For pseudopostcodes, we estimated frequencies based on national data for homelessness and then measured rates empirically. Pseudopostcodes cover overseas addresses and “unknown” plus “no fixed abode” (NFA) status [70]. ZZ99 3VZ is the postcode indicating NFA, and in its sector, ZZ993, there are nine recognized pseudopostcodes [70], with others representing whole countries including England, Wales, and Guernsey (e.g. for visitors given pseudocodes instead of their full postcode). A national estimate of the NFA postcode frequency is derivable as follows. In the UK, in 2020, there were ~27.8 M households with a mean size of 2.4 [80]. Of the UK population, 84.3% live in England [81]. In 2020, ~11.4% of the 68,180 households in England who were homeless or threatened with homelessness were of NFA and about two-thirds were single households [82]. Thus, the proportion of people who are of NFA could be estimated as (11.4% × 68,180 × [^2^/_3_ × 1 + ^1^/_3_ × 2.4]) ÷ (84.3% × 27.8 × 10^6^ × 2.4) = 0.0203%. However, empirically, rates were an order of magnitude higher in our healthcare context. In the SystmOne database, for people with valid NHS numbers and postcodes (one postcode per person; *n* = 612,056), 2336 (0.382%) had a pseudopostcode (defined as starting ZZ99), 2250 (0.368%) had a ZZ993 sector, and 1232 (0.201%) had a NFA postcode. We used 0.201% as the estimate of *p*_*pseudopostcode_unit*_, and 2250/1232 = 1.83 as the estimate of *k*_*pseudopostcode*_.

#### Empirical discrepancy(error) rates

We report, using anonymous Structured Query Language (SQL) queries, some empirical estimates of error rates for the linkage between the two largest databases, RiO and SystmOne. Original records were linked by NHS number and counted by discrepancy type (e.g. DOB mismatch; DOB off-by-one error). We did not perform NHS number checksum validity checks for these queries, but eliminated test NHS numbers. We also report metaphone match probabilities. Name comparisons in SQL removed spaces and were case-insensitive; name/metaphone comparisons for this purpose via Python also removed punctuation and, for metaphones, accents (due to limitations of the metaphone package), giving rise to very slight discrepancies with SQL-based totals.

The error rate priors derived from these analyses represent an example of limited information derived from known match status (see Table 2) that might confer an advantage on linkages involving the database pair involved. For all other database pairings, no prior information was available to the system about discrepancy rates, or any other information dependent on knowing the true match status for any records.

#### Inclusion and exclusion criteria

For Bayesian linkage tests, we included people known to CPFT via any of the source databases, excluding people without a known NHS number (since NHS numbers were used as the gold standard for linkage verification), whose NHS number was invalid (by checksum or because it was in the official test range starting 999), or without a known DOB (as a practical restriction rather than a requirement). NHS numbers are not a perfect standard for personal identity—for example, overseas nationals requiring emergency healthcare may not yet have one, and people changing gender in the UK may apply for a fresh NHS number [83]—but they are a very good one, being assigned at birth to everyone in the UK and being highly unlikely to change.

#### Data extraction, de-identification, and linkage

The following information was extracted from each database separately, for each patient with an NHS number recorded. (1) For matching we extracted forename(s), surname, gender, DOB, and all known postcodes with associated start/end dates if available (excluding postcodes in an invalid format) [84]. Each data set was hashed using the new system described above, removing all direct identifiers. We used a single step for extraction and hashing, removing the need to store identifiable information on disk even transiently. (2) For validation, we extracted a hashed (encrypted) NHS number, to enable linkage verification, without using direct identifiers. (3) For bias assessment, we extracted gender (again); ethnicity (coded Asian, Black, mixed, White, other, unknown) [85]; DOB blurred to the first of the month; age of first referral to MH services (as far as could be ascertained within each system, in integer years, from the first referral date minus the DOB); the last available Index of Multiple Deprivation (IMD, derived originally from postcode); whether a World Health Organization International Classification of Diseases (tenth revision) (ICD-10) diagnosis had been coded; whether an ‘F’ (mental disorder) ICD-10 diagnosis had been coded; and whether a severe mental illness (SMI) ICD-10 diagnosis had been coded. We defined SMI as a lifetime coded diagnosis of F20* (schizophrenia), F21* (schizotypal disorder), F22* (persistent delusional disorder), F31* (bipolar affective disorder), F32.2*/F32.3* (severe depressive episode), or F33.2*/F33.3* (recurrent depressive disorder, severe), where ‘*’ is a wildcard, following the UK National Institute for Health and Care Excellence definition [86]. We expressed IMD as a deprivation centile, from 0 = least deprived in England (IMD#32,844) to 100 = most deprived (IMD#1) [87], calculating the centile of IMD sequence numbers, i.e. not correcting for population; compare [3].

The settings for the de-identification and linkage system are shown in Table 2. We attached de-identified validation and bias measures as “optional extra” information, in addition to that used for linkage itself, as described above.

#### Comparison between pairs of databases

We linked databases in pairs, using only de-identified data. For example, we used CDL patients as probands against the RiO data set, and vice versa. We used an additional validation option in our software that included the (encrypted) ID of the leading candidate even if there was no clear winner, to examine the effects of varying match thresholds. We also linked databases to themselves, to estimate best-case matching performance.

#### Accuracy of matching

A given proband from the first database is either present or absent in the sample from the second database. The matching system involves both detecting the existence or non-existence of the proband in the sample, and identifying the correct candidate. We examined the first aspect of performance using standard signal detection theory (SDT) methods [88]. We defined a hit (true positive, TP) if the proband was present in the sample and the system declared a match. We defined a correct rejection (true negative, TN) if the proband was absent in the sample and the system did not report a match. We defined a miss (false negative, FN) if the proband was present in the sample but the system did not detect the match (including if it could not resolve two closely matching candidates so declared no winner). We defined a false alarm (false positive, FP) if the proband was absent from the system but the system declared a match (inevitably, to an incorrect candidate). In practice, FPs are likely substantially worse from a research perspective than misses. We calculated the true positive rate (TPR; hit rate, sensitivity, recall) = *P*(match declared | proband in sample); true negative rate (TNR, specificity) = *P*(no match declared | proband not in sample); false positive rate (FPR, 1 − specificity) = *P*(match declared | proband not in sample); and false negative rate (FNR, miss rate, 1 − sensitivity, 1 − recall) = *P*(no match declared | proband in sample). Additionally, we calculated the misidentification rate (MID): *P*(person misidentified | match declared). Note that this two-stage method differs slightly from some others’ approaches; see Appendix 2 and Supplementary Table 1 for rationale and comparison.

We examined the effects on these measures of varying the thresholds *θ* and *δ* (range 0–15 in steps of 1). Sensible defaults are required as end users are likely to require de-identified linkage without the possibility of gold-standard verification. We established values of *θ* and *δ* that minimized a weighted performance metric WPM = FNR + *w*_*MID*_⋅MID for non-self database comparisons, setting *w*_*MID*_ = 20 to reflect a preference for accuracy over comprehensive linkage; we provide these values of *θ* and *δ* and used them as defaults.

#### Bias relating to demographic factors and psychiatric diagnosis

We measured the effects of bias according to factors known about the proband, namely birth year; sex/gender (male, female, other/unknown); ethnicity; deprivation centile; diagnostic groupings (SMI coded, ICD-10 MH code excluding SMI recorded, no MH code recorded). We did not use age at first contact with MH services as a predictor, as it was strongly anticorrelated with birth year; compare [4].

For this purpose, we used the RiO database as probands, as it was the best characterized in terms of mental health diagnostic coding and the second best for ethnicity coding (see Results), and the SystmOne database as the sample, since this was the largest and broadest (see Results). We restricted to probands known to be in the sample, and with demographic information categorizable as above (i.e. excluding those with no recorded postcode and thus no deprivation centile known). We used a binary dependent variable of correct linkage (declaring a match and to the correct person), calculated using default thresholds, and predicted it via logistic regression.

#### Mechanistic reasons for linkage failure

We established categories of linkage failure reason for the RiO → SystmOne pair, as this gives an indication of where our system could most usefully be improved. Using only de-identified data, we examined probands who should have been matched but were not matched at the software’s default thresholds (including misses and misidentifications), and compared each to their corresponding true match (themself) in the sample. This established the proportion of failed linkages in which forenames differed, DOBs differed, and so forth.

### Results

#### Population frequencies

Random matches on the first two characters of a name (F2C) were commoner than random matches on metaphone. From the US forename/surname frequency data, we found that the probability of two randomly selected people sharing a metaphone was 0.0124 (surname) or 0.00503 (forename); the probability of sharing a metaphone but not a name was 0.000842 (surname) or 0.00259 (forename); and the probability of sharing a metaphone but not a name or F2C was 0.000633 (surname) or 0.00147 (forename). Similarly, the probability of sharing a F2C was 0.0221 (surname) or 0.0185 (forename); the probability of sharing a F2C but not a name was 0.0105 (surname) or 0.01602 (forename); and the probability of sharing a F2C but not a name or metaphone was 0.0103 (surname) or 0.0149 (forename). (These are mean probabilities across the population; for the matching algorithm, we used name-specific probabilities instead.) These data contributed to the decision to use metaphone as the more specific first partial match for names, and F2C as the less specific second partial match.

In the subsample of people paired with all others in the subsample except themselves (see Methods), there was a birth year range from 1906–1996, but *p*_*f*_^*DOB*^ = 9.03 × 10^−5^, equivalent to *b* = 30.3; we therefore used *b* = 30 for validation (Table 2). The empirical value of *p*_*pnf*_^*DOB*^ was 0.00631, very close to the expected value of 0.00630 for this value of *b*.

Other population frequencies used for validation are summarized in Table 2.

#### Subjects

Subject counts and demographics for all databases are shown in Table 3. Across all databases, there were 756,821 distinct people with NHS numbers recorded. Databases varied in size and in aspects of coding. The SystmOne database was the largest; the PCMIS and RiO databases were the best characterized in terms of MH data, with the RiO database having the best representation of those with SMI codes recorded. Coding errors were apparent even in summary data, such as DOBs in the future (Table 3), making cross-comparisons a realistic challenge.

**Table 3:** Characteristics of the source databases for validation. #, number. ‖ Data from anonymous queries of source databases prior to per-patient de-identified data extraction. ‡ Evidence of under-coding. ¶ Measured during tests where database queries and hashing were a separate step; for final analysis, data were extracted and hashed in a single step. † Indicates evidence of some coding errors (e.g. DOB in the future). * Categories combined for accuracy analysis. Abbreviations: “ < 10” small-group suppression applied; CTV3, Clinical Terms Version 3; ICD-10, World Health Organization International Classification of Diseases, tenth revision; k, thousand; MH, mental health; SD, standard deviation; y, year

Property	Database			
	CDL	PCMIS	RiO	SystmOne
**NATURE**				
Nature and dates	Secondary care MH services (> 10 k referrals/year from 1999–2012)	Psychological therapy services (> 1 k referrals/y, 2008–20, > 10 k referrals/y, 2015–)	Secondary care MH services (> 10 k referrals/y from 2012–21)	Community services (> 10 k referrals/y from 2007–); secondary care MH services (2020–). Live link to the NHS Spine, likely to improve validation of identifiers
Middle names extracted	None recorded	One	Up to four (aliases etc. also recorded but only “usual name” used here)	One (including some single-character names i.e. likely initials)
Postcode handling	Current	Current and previous	Dated history	Dated history
Principal coding system	ICD-10	ICD-10	ICD-10	Read/CTV3
**SIZE**				
‖ Total number of people	162,874	120,966	216,739	619,062
‡‖ Number with no DOB	0	0	352	< 10
Number included (valid NHS# + DOB)	152,888	117,961	208,632	613,169
† Duplicated NHS numbers: #records (#distinct NHS numbers duplicated)	0 (0)	6,356 (3,142)	0 (0)	0 (0)
**SOFTWARE PERFORMANCE**				
¶ Time to hash identity file (s)	56	41	138	328
Time to link to self (s)	608	544	1259	5437
Time to link to next (s)	→ PCMIS, 605	→ RiO, 903	→ SystmOne, 2651	→ CDL, 1151
**DEMOGRAPHICS**				
**Year of birth**				
† Range (years)	1890–2012	1915–2049	1902–2021	1899–2022
Mean ± SD (years)	1963 ± 26	1979 ± 15	1973 ± 24	1974 ± 29
**Sex/gender**				
Female (%)	55.4	63.9	55.4	54.1
Male (%)	44.6	35.5	44.6	45.9
* Other (%)	0.0007	0	0.03	0.004
‡* Unknown (%)	0.003	0.6	0.01	0.02
**Ethnicity**				
Asian (%)	0.86	2.47	1.56	3.12
Black (%)	0.37	0.90	0.84	0.88
Mixed (%)	0.41	1.97	1.42	1.02
White (%)	54.47	74.08	57.18	39.70
Other (%)	0.79	1.53	1.01	1.53
‡ Unknown (%)	43.10	19.05	37.99	53.75
**Coded ICD-10 diagnoses** ‡				
Severe mental illness (%)	2.65	0.35	2.95	0.18
MH (‘F’) code but no SMI (%)	5.89	88.76	16.67	0.62
* Code but no MH code (%)	1.68	0.34	0.77	0.03
* No ICD-10 codes (%)	89.78	10.56	79.61	99.17
**Address information**				
Postcodes per person: mean (range)	0.992 (0–1)	0.972 (0–2)	1.22 (0–20)	0.998 (0–1)
**Deprivation centile (0 least, 100 most)**				
Range	0.027–100	0.12–99.7	0.015–100	0–100
Mean ± SD	43.5 ± 26.9	41.1 ± 26.2	44.7 ± 27.1	48.6 ± 27.4
Unknown (%)	0.9	6.9	1.1	2.1
**Age at first MH care**				
† Range (years)	0–115	−39–97	−10–113	−1–105
Mean ± SD (years)	43.5 ± 26.1	36.7 ± 14.9	39.1 ± 24.1	37.5 ± 21.9
Unknown (%)	0	0.0017	42.8	85.7

#### Empirical discrepancy rates: dates of birth

Of people linked between the RiO and SystmOne databases purely by NHS number (*n* = 126,904), no DOBs were absent. DOBs, which cannot change veridically, did not match in 0.492% of people. Of DOB mismatches, 93.3% were “single component” mismatches (day mismatch but same month/year 43.9%, month mismatch but same day/year 27.6%, year mismatch but same day/month 21.8%). Of DOB mismatches, 30.9% were out-by-one errors (day out by one but same month/year, or month out by one but same day/year, or year out by one but same month/day) and 14.7% were out-by-two errors. Digit transposition errors (day digits transposed, month digits transposed, or last two digits of year transposed) accounted for 2.7% of DOB mismatches, and day/month transposition errors (e.g. United States versus UK format errors) accounted for 0.48% of DOB mismatches. Where DOBs did not match, the mean absolute difference in date was 772 days. Thus, DOB errors were fairly rare and most were accounted for by single-component errors, but we elicited no further dominant category beyond that. We took 0.00459 (93.3% × 0.492%) as *p*_*ep*_^*dob*^ (Table 2). Because complete DOB mismatches were rare (6.7% × 0.492% = 0.00033), and because there are major computational efficiencies in being able to “shortlist” by DOB, we took *p*_*en*_^*dob*^ = 0 (Table 2). As set out in the Methods, we did not test linkage for those few people in one database with a missing DOB (Table 3), but we note that with our default settings (Table 2), an exact DOB match adds a substantial LLR of +9.3 (with starting odds of about −13.7).

#### Empirical discrepancy rates: names

Other identifiers may change veridically as well as through error. For the same linkage, the primary forename (“usual” name, where specified, stripped of spaces and compared in case-insensitive fashion) differed for 2992/126,884 people (2.36%), and surname differed for 6166/126,904 people (4.86%). Both differed for 560/126,884 (0.44%), and forename/surname were different and transposed for 98/126,884 (0.077%). Thus, overall, name discrepancies were relatively common. When restricted to people of M/F gender and whose gender was identically recorded in both databases, forename mismatches occurred in 2.37% of females (1727/72,948) and 2.16% of males (1153/53,474), a small but significant difference (*χ*^2^_1_ = 6.0909, *p* = 0.01359), while surname mismatches occurred in 6.78% of females (4947/72,958) and 2.22% of males (1185/53,484), a highly significant difference (*χ*^2^_1_ = 1392.7, *p* < 2.2 × 10^−16^). We did not analyse genders other than M/F as numbers were very small and there was no consistent matching (e.g. an “unknown” code in one database and an “intersex/other” code in another database, or M/F in one database and an unknown or intersex/other code in the other). We therefore used separate error rates by gender.

For forenames, the probability of a metaphone match but not a name match (via the Python process, described above) was F 652/72,948 (0.894%), M 449/53,474 (0.840%), taken as *p*_*ep1*_^*forename*^ (Table 2); that of a F2C match but not a name or metaphone match was F 643/72,948 (0.881%), M 368/53,474 (0.688%), taken as *p*_*ep2np1*_^*forename*^; and that of no match at all was F 417/72,948 (0.572%), M 334/53,474 (0.625%), taken as *p*_*en*_^*forename*^.

For surnames, similarly, we took *p*_*ep1*_^*surname*^ as F 402/72,958 (0.551%), M 252/53,484 (0.471%); *p*_*ep2np1*_^*surname*^ as F 276/72,958 (0.378%), M 132/53,484 (0.247%); *p*_*en*_^*forename*^ as F 4140/72,958 (5.67%), M 719/53,484 (1.34%).

Forename transposition rates did not differ significantly by gender. We examined people matched by NHS number and restricted to those who had at least two distinct forenames in a notional sample database (SystmOne) and at least one forename in a notional proband (RiO) database. Of these, the proportion deviating from strict name ordering (that is, for whom sample forename 2 = proband forename 1, or sample forename 1 = proband forename 2 if present), was F 90/54,480; M 91/41,736 (*χ*^2^_1_ = 3.24, *p* = 0.0719), for those with matching gender. We took the probability across all matched people, 184/96,569 (0.191%), as *p*_*u*_^*forename*^ (Table 2).

#### Empirical discrepancy rates: gender

Gender was known but mismatched in 0.33% (taken as *p*_*e*_^*gender*^; Table 2). If only male and female categories were included, these mismatches fell to 0.29%.

#### Empirical discrepancy rates: postcodes

For postcodes, we restricted to people with exactly one postcode known to each database (*n* = 101,349), to reduce the possibility that differences represented a home move, or occasionally a change in the postcode system itself, rather than an error, though this possibility is far from eliminated. (We refer in general to errors or discrepancies, encompassing mismatches for any reason.) Postcodes differed for 31.0% of these people. Postcode units differed but the sectors matched for 0.97% of people (taken as *p*_*ep*_^*postcode*^; Table 2), and thus there was a complete mismatch for 30.0% of people (taken as *p*_*en*_^*postcode*^; Table 2). For 0.027% of people, postcodes matched only if the last two characters were transposed.

#### Linkage speed

The comparison process was fast enough to be readily achievable in a short period of time for real-world databases. Hashing speed, and selected linkage speeds for databases being compared to themselves, are shown in Table 3. The largest pair was linked in 2651 s (44 min).

#### Matching performance

**Fig. 4:**
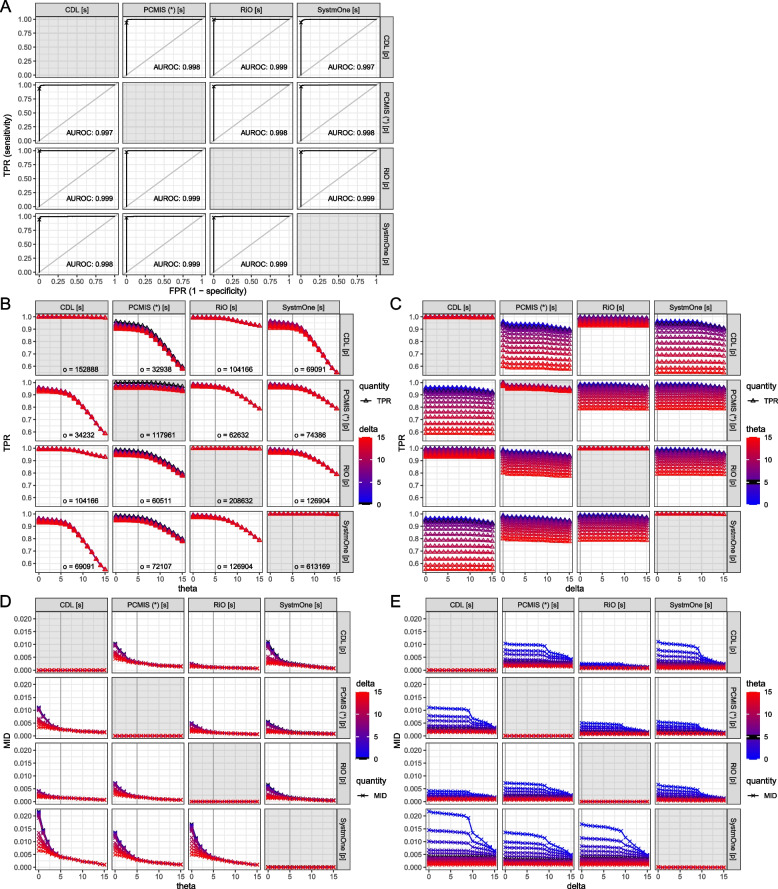
Matching performance for pairwise linkages between medical records databases, at different decision thresholds. In each panel, rows show the “from”/source/proband [p] database, and columns show the “to”/destination/sample [s] database (see Table 3). **A** Performance based on calculated log odds only: receiver operating curves using the software’s calculated log odds as the predictor (ignoring *δ*, i.e. taking *δ* = 0). The response variable was whether the proband was in the sample. (This does not guarantee that the correct candidate has been identified, for which see panels D, E.) Crosses indicate the default value of *θ*; diagonal lines represent random classification; AUROC, area under the receiver operating curve. Plots are not shown for databases matched to themselves, for which FPR is not calculable since all probands are in the sample. **B**,** C** True positive rate (TPR, declaring a match when the proband is in the sample, regardless of whether the correct person is identified), based on the two-stage decision process using *θ* and *δ*. Note the non-zero baseline, and that the TPR can include misidentification (see Methods). Panel B plots against *θ*, the minimum log odds for a match to be declared. The number of people overlapping between the two databases, *o*, is shown. Note that PCMIS TPR values are lower when it is a sample database than a proband database, as it contained NHS number duplication (see Table 3); a priori, this might reduce the TPR slightly when this database is the sample. Panel C plots against delta, *δ*, the additional log-odds threshold by which the leading candidate must beat the next-best candidate for a match to be declared. **D**,** E** Misidentification rates (MID): the probability that a declared match was for the wrong person. Note the difference of scale. Graphical conventions as for B, C. Vertical lines and black segments on the colour spectra show the software’s default thresholds

Figure 4 shows matching performance for the pairwise linkages. Log odds predicted the presence of the proband in the sample with area under the receiver operating curve (AUROC) values in the range 0.997–0.999 for non-self database comparisons (Fig. 4A). As expected, there was a trade-off between detection accuracy and misidentification rates (Fig. 4B–E). Increasing *θ* reduced the TPR (Fig. 4B), as expected, but also the MID (Fig. 4D). The effect of *δ* on the TPR was relatively small at any given value of *θ* (Fig. 4C), though increasing *δ* did reduce the TPR, particularly at low levels of *θ*. Increasing *δ* substantially reduced the MID if *θ* was low, but had little effect if *θ* was high (Fig. 4E). The fact that the PCMIS database contained duplicate records was apparent in that it was the only database to have a TPR noticeably below 1 when linked to itself for *δ* > 0 (Fig. 4C).

Taking mean performance metrics across all non-self database comparisons, we found that TPR, accuracy, F1 score, and distance to the receiver operating curve (ROC) corner were all optimized at *θ* = *δ* = 0, the lowest values tested. In contrast, MID and FPR were optimized at *θ* = *δ* = 15, the highest values tested. WPM was optimized at *θ* = 5, *δ* = 0, which we used as defaults. Table 4 summarizes pairwise linkage performance at those defaults. Overall, for non-self linkages, the mean TPR at these default settings was 0.965 (range 0.931–0.994, including correct linkages and misidentifications) and the mean misidentification rate was 0.00249 (range 0.00123–0.00429).

**Table 4:** Summary of pairwise linkage performance at selected thresholds. (A) “Odds on”: performance at *θ* = *δ* = 0, for comparison to the 50% threshold (*p* = 0.5, log odds = 0) of reference [24]. These settings yield a high TPR, at the cost of some misidentification. (B) Performance at the software’s default thresholds of *θ* = 5 and *δ* = 0, which optimized a weighted performance metric favouring MID reduction over TPR (see text). (C) Performance at *θ* = *δ* = 15, for a low MID. Values are a subset of data from Fig. 4. TPR, true positive rate or recall (detection of a proband who was in the sample, including correct linkages and misidentifications); MID, misidentification rate (the proportion of probands incorrectly identified). Values shown to three significant figures. † Note that the PCMIS database contained records with duplicate NHS numbers (see Table 3), particularly relevant when it is the sample database

Proband database	Sample database			
	CDL	PCMIS †	RiO	SystmOne
**A. At *θ* = *δ* = 0 (high TPR):**				
CDL	TPR: 1.00; MID: 0.00000654	TPR: 0.961; MID: 0.0104	TPR: 0.996; MID: 0.00259	TPR: 0.964; MID: 0.0111
PCMIS	TPR: 0.959; MID: 0.0111	TPR: 1.00; MID: 0.00000848	TPR: 0.985; MID: 0.00503	TPR: 0.985; MID: 0.00554
RiO	TPR: 0.996; MID: 0.00414	TPR: 0.987; MID: 0.00722	TPR: 1.00; MID: 0	TPR: 0.990; MID: 0.00657
SystmOne	TPR: 0.963; MID: 0.0218	TPR: 0.986; MID: 0.0136	TPR: 0.990; MID: 0.0168	TPR: 1.00; MID: 0.000139
Mean (range) for non-self linkage	TPR: 0.980 (0.959–0.996) MID: 0.00965 (0.00259–0.0218)			
**B. At *θ* = 5, *δ* = 0 (software defaults, balanced performance):**				
CDL	**TPR: 1.00; MID: 0.00000654**	**TPR: 0.935; MID: 0.00314**	**TPR: 0.993; MID: 0.00123**	**TPR: 0.941; MID: 0.00276**
PCMIS	**TPR: 0.931; MID: 0.00279**	**TPR: 1.00; MID: 0.00000848**	**TPR: 0.970; MID: 0.00148**	**TPR: 0.972; MID: 0.00174**
RiO	**TPR: 0.994; MID: 0.00159**	**TPR: 0.973; MID: 0.00202**	**TPR: 1.00; MID: 0**	**TPR: 0.976; MID: 0.00171**
SystmOne	**TPR: 0.941; MID: 0.00429**	**TPR: 0.974; MID: 0.00328**	**TPR: 0.976; MID: 0.00387**	**TPR: 1.00; MID: 0.000139**
Mean (range) for non-self linkage	**TPR: 0.965 (0.931–0.994)** **MID: 0.00249 (0.00123–0.00429)**			
**C. At *θ* = *δ* = 15 (low MID):**				
CDL	TPR: 0.990; MID: 0	TPR: 0.577; MID: 0.00137	TPR: 0.924; MID: 0.000664	TPR: 0.549; MID: 0.000633
PCMIS	TPR: 0.588; MID: 0.00134	TPR: 0.928; MID: 0	TPR: 0.786; MID: 0.000609	TPR: 0.788; MID: 0.000699
RiO	TPR: 0.926; MID: 0.000673	TPR: 0.774; MID: 0.000619	TPR: 0.994; MID: 0	TPR: 0.788; MID: 0.000320
SystmOne	TPR: 0.550; MID: 0.00103	TPR: 0.777; MID: 0.00105	TPR: 0.787; MID: 0.000980	TPR: 0.997; MID: 0
Mean (range) for non-self linkage	TPR: 0.735 (0.549–0.926) MID: 0.000832 (0.000320–0.00137)			

#### Bias relating to demographic factors and psychiatric diagnosis

Linkage success was associated with several demographic factors (Table 5). Younger people (those with greater birth year) were slightly less likely to be matched correctly. (However, the number of postcodes recorded per person exhibited small positive correlations with birth year, ranging from *r* =  +0.0275 to *r* =  +0.0875 across databases.) Males were more likely to be matched correctly. People in the Black or mixed ethnicity groups were more likely to be matched correctly, but people with unknown ethnicity were less likely to be matched correctly, and there was no effect of Asian or “other” ethnicity. People living in more deprived areas were slightly less likely to be matched correctly. The presence of a pseudopostcode had a small simple effect to reduce the likelihood of linkage (by *Z* test of coefficient), but this effect did not persist over and above all others (by *F* test with type III sums of squares). The presence of MH ICD-10 codes was associated with a greater likelihood of linkage.

**Table 5:** Effects of demographic factors on linkage accuracy, for the RiO (proband) to SystmOne (sample) comparison. Logistic regression predicting correct linkage (declaring a match to the correct person) amongst probands known to be in the sample, excluding those without a known deprivation centile (final *n* = 126,179), at default decision thresholds of *θ* = 5 and *δ* = 0. Coefficient: change in log odds of linkage for every unit change in the predictor (positive coefficient, greater likelihood of linkage; negative coefficients, lesser likelihood).* F* tests are from analysis of variance using Type III sums of squares (“over and above” all other predictors).* Z* tests are simple tests of coefficients. All values are shown to three significant figures. *** *p* < 0.001; ** p < 0.01; * *p* < 0.05; ICD-10, World Health Organization International Classification of Diseases, tenth revision; MH, mental health; NS, not significant; SMI, severe mental illness

Term	*F*	*p*_*F*_	Coefficient	Standard error	*Z*	*p*_*Z*_
(Intercept)	–	–	+29.9	1.63	+18.4	< 2 × 10^−16^ ***
Birth year (≈ inverse age)	*F*_1,126166_ = 277	< 2.2 × 10^−16^ ***	−0.0133	0.000823	−16.2	< 2 × 10^−16^ ***
Sex	*F*_2,126166_ = 129	< 2.2 × 10^−16^ ***				
Female			(reference)			
Male			+0.618	0.0406	+15.2	< 2 × 10^−16^ ***
Other or unknown			−1.39	0.364	−3.81	0.000137 ***
Ethnicity	*F*_5,126166_ = 20.5	< 2.2 × 10^−16^ ***				
White			(reference)			
Asian			+0.0910	0.147	+0.617	0.537, NS
Black			+ 0.564	0.262	+2.16	0.0312 *
Mixed			+0.506	0.175	+2.89	0.00392 **
Other			+0.0618	0.208	+0.297	0.767, NS
Unknown			−0.340	0.0387	−8.79	< 2 × 10^−16^ ***
Deprivation centile (0 least, 100 most)	*F*_1,126166_ = 17.4	3.02 × 10^−5^ ***	−0.00287	0.000681	−4.21	2.54 × 10^−5^ ***
Diagnostic group	*F*_2,126166_ = 76.7	< 2.2 × 10^−16^ ***				
No MH ICD-10 codes			(reference)			
MH code but not SMI			+0.672	0.0627	+10.7	< 2 × 10^−16^ ***
SMI			+0.779	0.141	+5.52	3.40 × 10^−8^ ***
Presence of a pseudopostcode	*F*_1,126166_ = 3.70	0.0544, NS	−1.03	0.461	−2.24	0.0249 *

#### Mechanistic reasons for linkage failure

Among people in the RiO → SystmOne pair who were not correctly linked at default thresholds (*n* = 3058 comprising misses and misidentifications), DOB matched fully in 94.5%, exhibited a partial match only in 4.09%, and was mismatched in 1.37%. Gender was correct in 97.3%, mismatched in 2.65%, and missing (on at least one side) in 0.0327%. The first name matched in 73.1%, exhibited a metaphone partial match (but no better) in 4.84%, exhibited a F2C partial match (but no better) in 7.13%, was mismatched completely in 14.8%, and was missing in 0.131%. Surnames exhibited a full match in 31.3%, a metaphone partial match in 2.22%, an F2C partial match in 1.77%, a complete mismatch in 64.7%, and there were no missing values. Postcodes exhibited at least one full match in 10.3%, at least one postcode partial match (but no better) in 14.0%, and complete mismatch in 75.0%, with missing values in 0.621%.

## Discussion

### Summary and algorithm performance

We developed a Bayesian method for linking records relating to people based on personal identifiers (forenames, surnames, DOB, gender, UK postcodes) that can deal with a variety of error types and operate either with direct identifiers or de-identified (hashed) versions. We validated this by linking multiple independent but overlapping electronic health records databases in an NHS Trust. Calculated log odds predicted probands’ presence in the sample database well, with AUROC 0.997–0.999 for comparisons between different databases. Decision threshold defaults were chosen to penalize misidentification over linkage failure 20-fold. By default, complete DOB mismatches were disallowed for computational efficiency. At these defaults, for comparisons between different databases, the mean probability of a proband being correctly declared to be in the sample was 0.965 (range 0.931–0.994), and the misidentification rate was 0.00249 (range 0.00123–0.00429). Performance was satisfactory despite the use of an interpreted programming language, with a linkage of 217 k people to 619 k people (1.34 × 10^11^ possible comparisons, with DOB pre-filtering reducing that to an estimated 8.6 × 10^8^) taking 44 min.

### Security of de-identified representations

The de-identified linkage files contain irreversibly hashed identifiers (e.g. DOB) and irreversibly hashed “fuzzy” versions of identifiers (e.g. year and month of DOB), with accompanying frequencies. Frequency representations are rounded and a floor applied, to avoid a 1:1 mapping between identifier and frequency. If start/end dates are provided (e.g. for postcodes), they are reproduced as plaintext in the output, but these are not direct identifiers. Gender is trivial to attack because gender is a three-state space whose frequencies are public knowledge, but gender by itself is not identifying, even for gender X. Assuming the user does not actively elect to add identifiable information via the “other information” feature (e.g. for linkage validation), the resulting linkage files contain nothing that would allow a human to re-identify a data subject by inspection.

Two re-identification attack methods should be considered. The first is that if the organizations leak their shared secret hash key, an attacker with access to a linkage file could re-identify some aspects by exhaustively hashing the entire space of possible identifiers—such as by hashing all DOBs in a 100-year period, or all names from a public name dictionary. This is a known problem with all such similar schemes, and the standard mitigation is of careful attention to the security of both keys and linkage files.

The second is that weak information is of course provided by the frequencies themselves (e.g. a high-frequency surname is more likely to be SMITH than MOZART) and the combination of multiple such data points might support a sophisticated jigsaw attack. The incorporation of frequency information aims to improve Bayesian accuracy but any frequency information increases vulnerability to cryptographic frequency attack (see Introduction); however, this may often be unimportant when the objective of de-identified linkage is simply to remove direct identifiers rather than to pass strong anonymity tests, such as for linking data in trusted research environments where the data itself is person-level and thus potentially vulnerable to jigsaw attack even without linkage data [45–51]. The standard mitigation here is technical and cultural security around linkage files, even if de-identified. However, a more important factor mitigating both risks is that a linkage file contains *only* identity information; even a plaintext version would not reveal, for example, medical information about a person.

### Predictors of correct linkage

Linkage was affected by demographic factors in ways that were generally as expected (Table 5). Males were more likely to be matched correctly than females; this is an expected effect [4], and we interpret it as a consequence primarily of the higher rate of surname discrepancies amongst females (observed empirically, as above). In turn, in our whole-lifespan cohort, this reflects the local cultural norm of women being more likely than men to change surname at marriage [89]. Indeed, complete surname mismatch was very common among people not correctly linked (64.7%). The association of X/unknown gender with worse linkage is obvious in that lack of gender information removes an informative identifier and gender X is associated both with change in gender status and with problems in electronic systems that only permit M/F recording.

Younger people (those with greater birth year) were slightly less likely to be matched correctly. This might reflect a number of potential causes, such as forename frequencies not matching our reference public dataset, or an association between age and accuracy of recording. It did not appear to be due to an “accumulation” of postcodes (and thus points of identification) with age, as the number of postcodes was positively, not negatively, correlated with birth year. Previous work with children [4] found varying effects of narrow age bands (e.g. 7–11-year-olds more likely, and 16–18-year-olds less likely, to be linked than infants); in contrast, our data encompassed a full age range.

People in the Black or mixed ethnicity groups were more likely to be matched correctly than those of white ethnicity, but people with unknown ethnicity were less likely to be matched correctly, and there was no effect of Asian or “other” ethnicity. These effects are quite similar to those seen by Downs et al. [4] prior to correction, but after adjustment they found that Asian, Black British/African, and “other” ethnic groups were less likely to be linked than the white/white British reference group, with no other effects. Previous work has found ethnicity minority status to be associated with more name recording errors [90, 91].

The association of worse linkage with pseudopostcodes is expected, as pseudopostcodes may indicate homelessness or visitor status. Both might be associated with fewer identifier verification opportunities, and the single national NFA pseudopostcode is a much less informative identifier than a true postcode (being shared by many more people). People living in more deprived areas were less likely to be matched correctly, over and above any pseudopostcode effect, which does not match some previous work finding better linkage in the most deprived quartile [4], though one possibility is that areas of higher deprivation are over-represented in our patient populations and thus high-deprivation postcodes become slightly less discriminating predictors of identity. Mechanistically, postcode mismatch was very common amongst those not correctly linked (75.0%), so another possible explanation is of a higher frequency of change of address among more deprived groups.

The association of MH diagnostic codes with better linkage is plausible, not least because more contact with MH services increases both the chance of diagnostic coding and the chance of accurate and complete recording of other details. Since SMI is associated with homelessness [92–94], it is worth noting that these effects were over and above those of pseudopostcodes. Others have similarly found better linkage to be associated with “any ICD-10 disorder” recorded in secondary care MH data [4].

### Comparison to other approaches

Our work was motivated by the need to link health data to external data sources, and an excellent demonstration of this is by Downs et al. [4], who linked health to the UK National Pupil Database (NPD), using direct identifiers (fully identifiably), via a mixed method beginning with an automated approach and finishing with manual checks. In this instance the NPD was expected to provide near-complete coverage, since it contains education data for all school pupils in England within a relevant time period [95], while the health data was from a London NHS Trust [4], and thus all probands (NHS) were expected to be in the sample (education). They linked 29,278/35,509 people (82.5%) successfully. Independent validation was not possible, so the misidentification rate after manual checking was assumed to be negligible. Our technique, albeit validated with health-to-health data, achieved mean TPR 96.5% and MID 0.249%. However, formal performance comparison would require checks using identical data [96].

There have been many linkage approaches in the non-Bayesian domain, including deterministic (rule-based) approaches [97], systems to cluster people automatically by identity to detect erroneous identifiers within a dataset containing overlapping identifiers [98], and non-Bayesian probabilistic methods [9, 99]. However, these do not address de-identified linkage.

There has also been significant prior work on Bayesian linkage (see Introduction). Notably, recent Bayesian linkage work for the large-scale SAIL Databank has used an SQL-based algorithm called MACRAL (Matching Algorithm for Consistent Results in Anonymised Linkage) to achieve sensitivity (TPR) > 94.6% and error rate < 0.2% [24]; this system outperformed several previous software packages. When using the same thresholds (their “50% threshold” or *p* = 0.5, equivalent to our *θ* = *δ* = 0), our system achieved a mean TPR of 98.0% (range 95.9%–99.6%) and a mean error rate of 0.965% (range 0.259%–2.18%). In contrast, performance at our default thresholds was numerically similar (Table 4). At default settings, self-linkage performance of our system exceeded, numerically, that of a large-scale de-identified study using Bloom filters [34]; however, valid performance comparison would require checks using the same data [96].

### Strengths and weaknesses

The primary strengths are that we provide a new technique for fully de-identified (or identifiable) linkage based only on common, non-unique personal identifier types, in portable cross-platform open-source software, and show that it has high accuracy. This may permit fully automatic linkage in situations where manual assistance is currently used, and permit fully de-identified linkages between organizations that do not share a common person-unique identifier (e.g. linking health data to education or social care data), improving IG, and requiring slightly less stringent regulatory burdens by eliminating identifiable data from the linkage pipeline. We also examine in detail the predictors of, and reasons for, non-linkage.

The primary weakness of fully de-identified linkage is that it is not verifiable intrinsically, only extrinsically. The proportion of probands left unlinked is readily apparent from the results, but how many are false negatives will be unknown; similarly, where people are linked, the proportion that are misidentified will remain unknown. We provide data to gauge how the software’s thresholds (*θ*, *δ*) influence error rates, which users can configure to their own preference regarding the relative costs of false negatives and misidentification, and the system yields an estimate of absolute linkage probability based on locally provided baseline priors. We also provide a full framework with which users can validate performance against their own data where gold-standard comparators are available, as we did in the present study.

Our system requires the user to extract data into a text-based file format for processing; this adds an extra step but the file formats supported are simple and the advantage is that the software can be used portably regardless of the nature of the source databases.

Our method uses domain-specific information, such as priors relating to name and DOB frequencies. The major alternative method is to estimate relevant probabilities directly from the data, such as via expectation maximization [5, 10, 42, 43, 99] or Bayesian estimation [5, 17–20]. Aspects of domain-specific priors might reasonably be expected to be stable over certain contexts. For example, the probability of unrelated people matching on DOB is likely to be approximately constant worldwide for a given age span; sex/gender ratios are likely to be similar and known accurately for a given country; name frequencies might be expected to be relatively similar e.g. across anglophone countries but not beyond (but may be readily derivable for any specific country as required); postcode frequencies apply to the UK but not elsewhere. The use of domain-specific information also allows strong priors about conditional dependence/independence, e.g. that forename frequency depends on gender, while DOB frequency does not. Additionally, the use of large-scale sources (e.g. large-country name frequency data) may confer an accuracy advantage over estimation from the data if the linkage data sets are quite small. However, although our system performed well (numerically) compared to other identifiable and de-identified probabilistic linkage systems (as above), and generalized very well to databases for which it had no prior information, we have not provided a head-to-head performance comparison with a system estimating frequency priors from the data, or head-to-head comparisons with other systems using identical data [96], which is a limitation of the present work.

It is harder to estimate the consistency of domain-specific error rates, such as name or DOB recording errors. Our rates (Results; Table 2) are similar to some previously estimated values [97, 99] but these do vary, including by institution [97]. Our system generalized well from the database pair used to estimate local error rates to all others (Table 4), though error rates within a single institution are likely to be somewhat correlated across databases by virtue of involving some of the same staff and procedures, even though some databases were temporally non-overlapping and the source systems varied in their automatic error-detection facilities (Table 3). However, in the de-identified situation where gold-standard validation (and thus the accurate measurement of local error rates) is not possible, defaults derived from a specimen medium-scale context may nonetheless be useful. As set out above, these may be overridden by accurate values for a given data set if available.

Some priors were derived from empirical discrepancy rates from one database pair (RiO/SystmOne), which might have conferred an advantage on that pairing if those discrepancies were not mirrored elsewhere; however, that pairing was not even the one with the best linkage (Table 4). Other priors (e.g. population size) were specific to our validation context, but are readily configurable for others’ contexts. More generally, the fact that linkage performance was good for those pairings for which no prior information at all was provided (Table 4) suggests that the system can generalize to at least some novel contexts where no information on true match status is available (not even information for a limited subset). However, that generalization may worsen outside of health care environments similar to ours, and independent validation for other settings would be desirable.

Our system provides user-configurable minimum values for name frequencies and user-configurable rounding for all frequencies. This is a trade-off provided to ensure de-identified representations are less likely to contain unusual or unique values, and to reduce spurious matches for extremely rare names, but both could reduce the accuracy of probability calculations. We report empirical validated accuracy at default settings. Security considerations around frequency information are discussed above.

Not all categories of error were explicitly represented. We attempted to model, and thus improve linkage despite, a number of “incomplete” error types, including name alterations (alterations that remained matched on metaphone or on first two characters; acceptance of accent transliterations; various forms of name re-ordering and name component reordering), DOB component alterations, and incomplete postcode errors, as well as complete mismatches in all identifiers. However, there were types of error we did not model (e.g. forename/surname transposition), and fuzzy representations that we did not use. Phonetic approximations, like the metaphones used here, are known to be worse for approximate matching than *n*-grams [100], but have the advantage of being a unitary representation readily suited to irreversible hashing. Future linkage systems might extend the methods developed here using richer approximation algorithms, such as the use of Bloom filters for approximate comparisons in the de-identified domain [34–36]. However, we note that surname mismatches were commonly associated with linkage failure and this problem might not yield simply to fuzzier approximations.

We prohibited “complete” DOB mismatches during validation, which prevented some linkages being made—0.033% of people in the RiO/SystmOne pair had a DOB mismatch that would have been eliminated by this pre-filtering (excluding those also ignored without a DOB), so this is one cap on current performance. To link large databases whilst permitting complete DOB mismatches is certainly possible using the current system, but re-implementation in a compiled language might be desirable for better performance [101].

Postcodes are of course a UK-specific address abstraction, but the principles of the system are readily adaptable to similar coding systems internationally, and we make our code freely available for use and adaptation.

## Conclusions

Fully de-identified matching with high accuracy is feasible and practical, even without a person-unique identifier and despite real-world errors and variations in data recording. We hope this method will facilitate fully automated linkage, subject to ethical and regulatory approvals and appropriate security measures, between organizations that do not share a common person-unique identifier, and do so in a way that is safer and more acceptable from an IG perspective than transient sharing of direct identifiers. A hybrid approach incorporating deterministic matching using person-unique identifiers, where available, would be expected to increase accuracy still further, and this is supported by our system. The software we developed to perform these tasks is freely available.

## Supplementary Information

**Additional file 1.**

## Data Availability

Source code is available via https://crateanon.readthedocs.io/ and https://github.com/ucam-department-of-psychiatry/crate. Raw de-identified data are not publicly available, under the terms of NHS Research Ethics approvals; for details of access and conditions, contact research.database@cpft.nhs.uk.

## Abbreviations

AUROC: Area under the ROC
CDL: Clinical Document Library
CPFT: Cambridgeshire & Peterborough NHS Foundation Trust
CRATE: Clinical Records Anonymisation and Text Extraction
CRS: Care Records System
CSV: Comma-separated value
CTV3: Clinical Terms Version 3
D: Data
DOB: Date of birth
F: Female
F2C: First two characters
FN: False negative
FNR: False negative rate
FP: False positive
FPR: False positive rate
H: Hypothesis
HMAC: Hash-based message authentication code
IAPT: Improving Access to Psychological Therapy
ICD-10: World Health Organization International Classification of Diseases, tenth revision
ID: Identifier/identity
IDX: Identity exchange
IG: Information governance
IMD: Index of Multiple Deprivation
JSON: Javascript Object Notation
JSONL: JSON Lines
k: Thousand
LLR: Log likelihood ratio
M: Male, or million
MACRAL: Matching Algorithm for Consistent Results in Anonymised Linkage
MD: Message digest
MH: Mental health
MID: Misidentification rate
NFA: No fixed abode
NHS: UK National Health Service
NPD: UK National Pupil Database
NS: Not significant
OA: (UK Office for National Statistics) Output Area
PCMIS: Patient Case Management Information System
PPI: Patient and public involvement
ROC: Receiver operating curve
SAIL Databank: Secure Anonymised Information Linkage Databank
SD: Standard deviation
SDT: Signal detection theory
SHA: Secure Hash Algorithm
SMI: Severe mental illness
SQL: Structured Query Language
TN: True negative
TNR: True negative rate
TP: True positive
TPP: The Phoenix Partnership
TPR: True positive rate
TTP: Trusted third party
UK: United Kingdom
WPM: Weighted performance metric
y: Year

## Footnotes

1 Working: Prior probability *P*(*H*) = ^1^/_100_; prior odds ^1^/_99_. *P*(*D* | *H*) = 1; *P*(*D* | ¬*H*) = ^1^/_99_; LR = 99. Posterior odds 1; posterior *P*(*H* | *D*) = 0.5.

2 Working: Prior probability *P*(*H*) = ^1^/_100_; prior odds ^1^/_99_. *P*(*D* | *H*) = 1; *P*(*D* | ¬*H*) = 0.001 [incorrect]; LR = 1000 [incorrect]. Posterior odds 10.1 [incorrect]; posterior *P*(*H* | *D*) = 0.91 [incorrect].

3 Working: Ignoring the possibility of error: prior probability *P*(*H*) = ^1^/_10_; prior odds ^1^/_9_. *P*(*D* | *H*) = 1; *P*(*D* | ¬*H*) = ^5^/_9_ (5 other Alices among 9 other people); LR = ^9^/_5_. Posterior odds ^1^/_5_; posterior *P*(*H* | *D*) = ^1^/_6_.

4 Working: Again, without errors: prior probability *P*(*H*) = ^1^/_10_; prior odds ^1^/_9_. *P*(*D* | *H*) = 0; *P*(*D* | ¬*H*) = ?; LR = 0. Posterior odds 0; posterior *P*(*H* | *D*) = 0.
