# Supplementary material for: De-identified Bayesian personal identity matching for privacy-preserving record linkage despite errors: development and validation
Source: BMC Med Inform Decis Mak. 2023 May 5;23:85. doi: 10.1186/s12911-023-02176-6 (PMC10163749; doi:10.1186/s12911-023-02176-6)
Supplement: Supplementary file 1 — Additional file 1. [file 12911_2023_2176_MOESM1_ESM.pdf]

SUPPLEMENTARY MATERIAL

De-identified Bayesian personal identity matching for privacy-preserving record linkage despite errors: development and validation

Rudolf N. Cardinal, Anna Moore, Martin Burchell, Jonathan R. Lewis (2023)

BMC Medical Informatics and Decision Making

TABLE OF CONTENTS

APPENDIX 1 2
APPENDIX 2 3
SUPPLEMENTARY TABLES 4
SUPPLEMENTARY REFERENCES 5

APPENDIX 1

If the proband were guaranteed to be in the sample, initial probabilities could be normalized to sum to 1, as follows. Probabilities could be calculated for each proband–candidate pair. They might initially be inaccurate in absolute terms, but their relative values would be meaningful. The constraint that the proband is in the sample gives $\Sigma_j P(H_j) = \Sigma_j P(H_j \mid D) = 1$ where j iterates over all candidates. For a given candidate i it would then be true that:

$$
\begin{aligned}
P(H_i|D) &= \frac{P(D|H_i)P(H_i)/P(D)}{\sum_{j=1}^{n_s} P(D|H_j)P(H_j)/P(D)} \\
&= \frac{\frac{1}{P(D)}P(D|H_i)\,P(H_i)}{\frac{1}{P(D)}\sum_{j=1}^{n_s} P(D|H_j)P(H_j)} \\
&= \frac{P(D|H_i)P(H_i)}{\sum_{j=1}^{n_s} P(D|H_j)P(H_j)}
\end{aligned}
$$

With equal priors, $H_i = H_j\ \forall i,j$ and this expression reduces to:

$$P(H_i|D)=\frac{P(D|H_i)}{\sum_{j=1}^{n_s} P(D|H_j)}$$

In log form, with natural logarithms,

$$\log P(H_i|D)=\log P(D|H_i)-\log\sum_{j=1}^{n_s} e^{\log P(D|H_j)}$$

or in pseudocode, *log_posterior_p_match[i] = log_likelihood[i] − logSumExp(log_likelihood)*.

However, as discussed in the main text, the assumptions behind this process are unlikely to hold in practice.

APPENDIX 2

The present classification problem can be approached in different ways using the framework of signal detection theory (SDT). When two databases are linked, if the proband (person from the first database) is not in the sample (second database) and no match is declared by the linkage software, that is typically defined as a correct rejection or true negative (TN). If the proband is in the sample and the software matches to the correct person, that is a hit or true positive (TP). If the proband is in the sample but no match is made, that is a miss or false negative (FN). If the proband not in the sample but the software finds a match anyway, which is necessarily an incorrect match, that is a false alarm or false positive (FP). These categories, TP, FP, TN, and FN, are the standard four categories of event in binary classification SDT (1).

That much is generally agreed, but there is a fifth possibility: the proband is in the sample, and the software matches to the wrong person. How should this be handled? In (2) and subsequently (3,4), this event is subsumed under the category of "false matches" and called a FP (**Supplementary Table 1**). Of course, that still makes for four mutually exclusive categories and is valid for many analyses. However, it violates an assumption of SDT (1) that the division into positive (P = TP + FN) and negative (N = TN + FP) events is a property of the "world", measurable entirely independently of the test and any decisions it takes (**Supplementary Table 1**). Thus, in this system, variations in the test's performance can affect measured "world" attributes such as prevalence, P/(P + N). In contrast, in the present study, we approach the question as two binary stages. The first, "is the proband correctly declared to be in the sample?", we characterize by standard SDT measures. The second, "if the proband is declared to be in the sample, is the correct person chosen as the match?", we characterize by the misidentification rate (**Supplementary Table 1**).

A further approach in line with SDT is to consider the "world" categories as {matches, non-matches} across all possible proband–candidate pairings, and the decision as {linked, unlinked} (5,6). Using this method, if a probands are compared to b distinct candidates, the prevalence is capped at $\min(a, b)/ab$.

SUPPLEMENTARY TABLES

Supplementary Table 1. Two different signal detection theory approaches to the present classification problem. Details are in **Appendix 2**. The five rows represent collectively exhaustive and mutually exclusive possibilities for a single proband being compared to a set (sample) of candidates. Differences between the two systems, shown in bold, relate to the handling of probands who are in the sample but who are misidentified. TP, true positive (hit); TN, true negative (correct rejection); FP, false positive (false alarm); FN, false negative (miss); N, actually negative (N = TN + FP); P, actually positive (P = TP + FN); PN, predicted negative (PN = TN + FN); PP, predicted positive (PP = TP + FP). In system 2, the misidentification rate is MID = m / PP = (b + d) / (b + d + e), the proportion of declared matches that identify the wrong person, which is the same as the false discovery rate (FDR) = 1 − positive predictive value = 1 − precision = FP / PP calculated using system 1. The TPR (recall) = TP / P calculated using system 1 must always be less than or equal to that calculated using system 2, as "system 1 TPR" = e / (c + e) ≤ "system 2 TPR" = (d + e) / (c + d + e). (In the special case that "system 2 TPR" is 1, c must be zero and therefore "system 1 TPR" must also be 1.)

**Event**	**Proband in sample?** (Property of world.)	**Match declared?** (Property of test.)	**Match is proband?** (Property of world + test.)	**Decision correct?** (Property of world + test.)	**Description**	**System 1** (2–4)	**System 2** (Present study.)
a	No	No, PN	–	Yes	Proband not in sample; correctly, no match is declared.	TN, N	TN, N
b	No	Yes, PP	No	No	Proband not in sample; software matches to a wrong candidate.	FP (false link), N	FP, N, *m*
c	Yes	No, PN	–	No	Proband is in sample; software fails to match.	FN, P	FN, P
d	Yes	Yes, PP	No	Identifying the proband is in the sample: yes. Finding the right person: no.	Proband is in sample; software matches to a wrong candidate.	**FP (false link), N**	**TP, P;** *m*
e	Yes	Yes, PP	Yes	Yes	Proband is in sample; software matches correctly.	TP, P	TP, P

SUPPLEMENTARY REFERENCES

1. Macmillan NA, Creelman CD. Detection Theory: A User's Guide. 2nd ed. Mahwah, N.J: Lawrence Erlbaum Associates; 2005. 492 p.

2. Karmel R, Gibson D. Event-based record linkage in health and aged care services data: a methodological innovation. BMC Health Serv Res. 2007 Sep 25;7:154.

3. Lyons RA, Jones KH, John G, Brooks CJ, Verplancke JP, Ford DV, et al. The SAIL databank: linking multiple health and social care datasets. BMC Med Inform Decis Mak. 2009 Jan 16;9:3.

4. Ferrante A, Boyd J. A transparent and transportable methodology for evaluating Data Linkage software. J Biomed Inform. 2012 Feb;45(1):165–72.

5. Blakely T, Salmond C. Probabilistic record linkage and a method to calculate the positive predictive value. Int J Epidemiol. 2002 Dec;31(6):1246–52.

6. Doidge J, Christen P, Harron K. Quality assessment in data linkage [Internet]. Office for National Statistics; 2021 [cited 2023 Feb 27]. Available from: https://web.archive.org/web/20221006134959/https://www.gov.uk/government/publications/joined-up-data-in-government-the-future-of-data-linking-methods/quality-assessment-in-data-linkage
